# Ethnoveterinary use of plants and its implication for sustainable livestock management in Nepal

**DOI:** 10.3389/fvets.2022.930533

**Published:** 2022-09-09

**Authors:** Yadav Uprety, Sangram Karki, Ram C. Poudel, Ripu M. Kunwar

**Affiliations:** ^1^Central Department of Botany, Tribhuvan University, Kathmandu, Nepal; ^2^Forest Research and Training Centre, Ministry of Forests and Environment, Kathmandu, Nepal; ^3^Nepal Academy of Science and Technology, Kathmandu, Nepal; ^4^Ethnobotanical Society of Nepal, Kathmandu, Nepal

**Keywords:** animal husbandry, *Cannabis sativa*, cattle health, ethnomedicine, useful plants

## Abstract

Traditional herbal remedies are used worldwide for treating both human and livestock health issues. Though such uses are relatively well-explored for humans, the ethnoveterinary uses of plant-based remedies in the healthcare choices of livestock in Nepal and associated knowledge are largely ignored. This is important as sustainable livestock production is an emerging issue. This study reviews the existing ethnobotanical studies conducted in Nepal and reports the use of 393 species of plants from 114 botanical families in ethnoveterinary practices. Thirty-four different ailments were treated using these plants. The present review revealed that Nepal has a rich diversity of ethnoveterinary plants. This study shows that traditional herbal medicine plays a significant role in meeting the livestock healthcare needs of Nepali farmers and hence is a viable practice. The study also contributes a wealth of knowledge about ethnoveterinary practices for further planning and use. This will provide an option for livestock farmers who cannot afford allopathic medicine or who are not allowed to use such medicine under organic farming schemes that are likely to be a part of sustainable livestock farming programs in Nepal soon.

## Introduction

Ethnoveterinary knowledge is deeply rooted in many traditional cultures and is an integral part of subsistence animal husbandry in many societies across the globe, including Nepal (1–4), India (5), Pakistan (6), Ethiopia (7), Romania (8), Spain (9), Switzerland (10), and elsewhere. Hailed as a traditional system and currently emerging as a scientific discipline, ethnoveterinary research, defined as “systematic investigation and application of veterinary folk knowledge, theory, and practice,” McCorkle (11) however, has little contribution to modern veterinary health sciences (12).

Animal rearing is the major occupation, the main source of livelihood, and a symbol of socioeconomic status for most of the population in Nepal. The livestock sector is one of the major contributors to the national Gross Domestic Product. According to the Ministry of Agriculture and Development, Government of Nepal (13), 87% of the country's population keeps some form of livestock at home; with 5.8 heads of livestock and poultry per household, Nepal has one of the highest ratios of livestock to humans in Asia (14). Nepal's livestock numbers are estimated to be 7.27 million cattle, 5.24 million buffaloes, 10.17 million goats, 0.8 million sheep, 48 million poultry, and 0.37 million ducks (15). This sector contributes around 11% to the national Gross Domestic Product and also has a high potential for growth (15).

Because of a lack of resources and modern veterinary facilities, most livestock raisers in Nepal practice herbal remedies to treat various livestock ailments. This could be an advantage rather than an issue for the livestock sector as there is a growing interest in organic livestock management. Herbal remedies may be considered one of the most important alternatives to treating organic livestock (16). The use of such low-cost medicinal plants is widespread among organic livestock raisers (17) and promoted for their sustainable use (16). As Nepal still practices traditional farming that relies on local resources and integrates crops and livestock, integrating ethnoveterinary practices into livestock management would be a sustainable approach. However, this needs to be integrated into the research, extension, and policy frameworks. Furthermore, as estimated by the Food and Agriculture Organization of the United Nations, the global demand for livestock-related products will increase by more than 50% by 2050, and the use of low-cost medicinal plants can contribute to even large-scale livestock farming. The research and policy interventions in this direction also contribute to food security and climate change mitigation.

The knowledge regarding the traditional use of plants in veterinary medicine has been preserved by practice and oral transmission as that of plant-based traditional medicines used for humans [e.g., (18, 19)]. Though these uses are relatively well-explored for humans, the ethnoveterinary uses of plant-based remedies in the healthcare choices of livestock in Nepal and associated knowledge are largely ignored. Many of the ethnobotanical studies conducted in Nepal have documented veterinary uses of medicinal plants along with documentation of medicinal plants used to treat human health issues contributing to the wealth of ethnoveterinary knowledge (1–3, 20, 21). Nevertheless, there are also a few studies particularly focused on ethnoveterinary uses of plants in Nepal [e.g., (4, 22, 23)].

In this paper, we reviewed the existing ethnobotanical studies conducted in Nepal and documented the knowledge of ethnoveterinary practices to appraise how traditional systems are associated with livestock health issues. The study also contributes to the wealth of knowledge of ethnoveterinary practices for further planning and use, as this will provide an option for livestock farmers who cannot afford allopathic medicine or who are not allowed to use such medicine under organic farming schemes that are likely to be a part of sustainable livestock farming programs in Nepal soon.

## Methods

### Data collection

We reviewed ethnobotanical studies published from Nepal in journals and proceedings from 1955 to 2022. As many of the ethnobotanical studies have also documented ethnoveterinary uses of plants, we first collected pertinent literature in Google Scholar, Scopus, ISI Web of Science, and Science Direct using specific keywords such as “medicinal plants,” “herbal medicine,” “ethnobotany,” “traditional knowledge,” “herbal practice,” and “Nepal.” Then, we used keywords such as “veterinary,” “animal(s),” “cattle” and “livestock” to find the species within the papers. However, as we saw that not always these terms were used in the source we also used keywords such as “goat,” “sheep,” “buffalo,” “cow,” “ox,” “poultry,” “duck,” and “yak,” as these are the common livestock in Nepal being managed in animal husbandry.

### Data analysis

Of the total 254 papers dealing with ethnomedicinal uses practiced in Nepal, we used 104 papers to prepare the master list of plant species used in ethnoveterinary. We further analyzed the papers into two categories—specific papers with a focus on ethnoveterinary and the general ethnobotanical papers having ethnoveterinary as one of the uses. The precision of botanical identification of species in this paper depended on that from original sources. Latin names and family names were verified in the Catalog of Life 2022 (https://www.catalogueoflife.org/). If the original name had been changed to another accepted name and was different from the original source we provided accepted names. We also provided a synonym for the accepted name if the accepted name in the original sources is now changed to a synonym. The master list of plants was further analyzed to categorize plants into different botanical families, growth forms, and parts used. The species associated with various ailments were also discussed. We followed Cook (24) to cluster plants according to the different ailment categories they help to cure.

## Results and discussion

### Ethnoveterinary studies conducted in Nepal

Of the total 254 papers on the ethnobotany of Nepal published from 1955 to date, 104 papers have contributed to the wealth of ethnoveterinary knowledge. The earliest ethnoveterinary use of the plant was found to be published in 1980 (1), though Sacherer (25) reported using poisonous plants for livestock in 1979. Of these 104 papers, only nine specifically dealt with ethnoveterinary (4, 22, 23, 26–31) while the rest were on general ethnobotany. Only a few of the publications reported information about herbal toxic effects, dosage, and conservation but the majority of the publications lacked this information, as also reported from Ethiopia (7). The records were reported from 54 districts out of 77 in Nepal. Of the total, 10 studies are reported from the Makwanpur district alone followed by Darchula (9), Kaski and Kavre (7 each), and Morang (6). These studies show that the animal husbandry in Nepal is largely intrigued by the ethnoveterinary practices. Furthermore, most of the studies cover the mid-hills of Nepal, where livestock holding size is highest (32) and the collection, use, and trade of medicinal plants are common (33).

### Ethnoveterinary plants: Diversity and distribution

**Figure 1:**
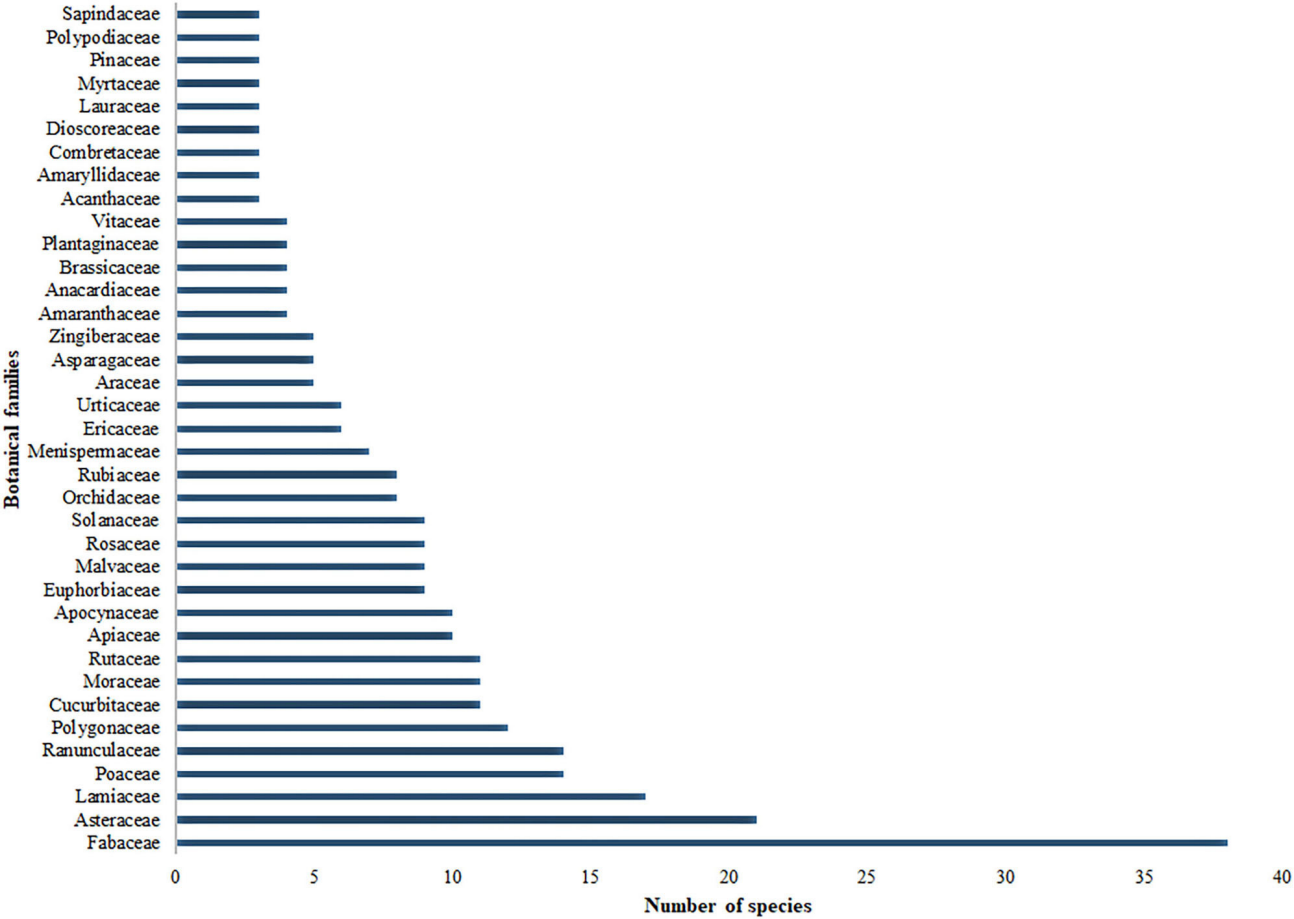
Dominant families with respect to species (families having three or more species are presented).

A total of 393 species belonging to 114 botanical families were reported to be used to treat different livestock ailments in Nepal (additional file). Angiosperms were represented by 102 families followed by Pteridophytes (five families), Gymnosperms (4), Fungi (2), and Lichens (1). Sixty-two botanical families were represented by single species, 13 families were represented by two species and 36 families were represented by three or more species. The largest family with 38 species was Fabaceae, followed by Asteraceae (21), Lamiaceae (17), Poaceae (14), Ranunculaceae (14), Polygonaceae (12), Cucurbitaceae (11), Moraceae (11), Rutaceae (11), Apiaceae (10), and Apocynaceae (10) (Figure 1). Species from the families Fabaceae, Asteraceae, and Lamiaceae are also the most important ethnoveterinary flora in Europe (16). Fabaceae is also reported to be one of the largest families in ethnoveterinary flora in Ethiopia (7). Likewise, Fabaceae and Lamiaceae are among the largest families of ethnoveterinary flora in South Asia (34) and Spain (35). Similarly, species from Asteraceae and Lamiaceae are reported to be extensively used in other studies that documented human use of medicinal plants (36, 37). *Ficus* (with seven species), *Delphinium* (6), *Prunus* (5), and *Bauhinia, Brassica, Persicaria*, and *Solanum* each with four species were the most common genera.

**Figure 2:**
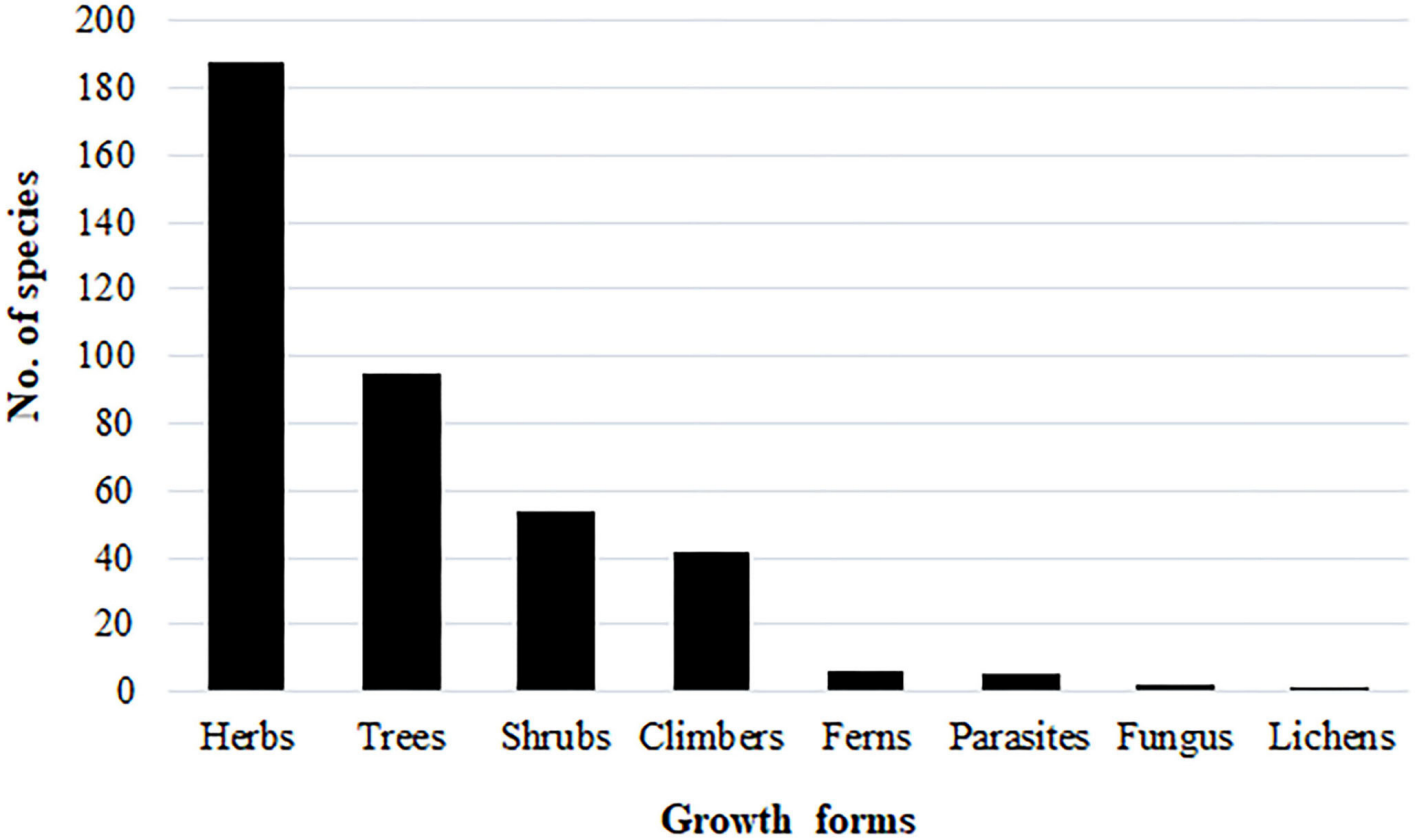
Species distribution according to plant growth forms.

In terms of growth forms, herbs were dominant with 188 species followed by trees (95) and shrubs (54) (Figure 2). In terms of plant parts used, the leaves were the most frequently used parts followed by roots, fruits/seeds, whole plant, bark, stem, rhizomes, oil/resin/latex, aerial parts, and flowers (Table 1, Supplementary Table). Leaves were also the most frequently used parts in ethnoveterinary in Namibia (38).

**Table 1:** Plant parts used and number of species.

**Parts used**	Number of species^*^
Leaves	127
Roots	86
Fruits/seeds	80
Whole plant	76
Bark	41
Stem/shoot	31
Rhizomes/tubers/bulbs	23
Oil/resin/latex	13
Aerial parts	13
Flowers	12

* Multiple parts were reported to be used for several species.

**Figure 3:**
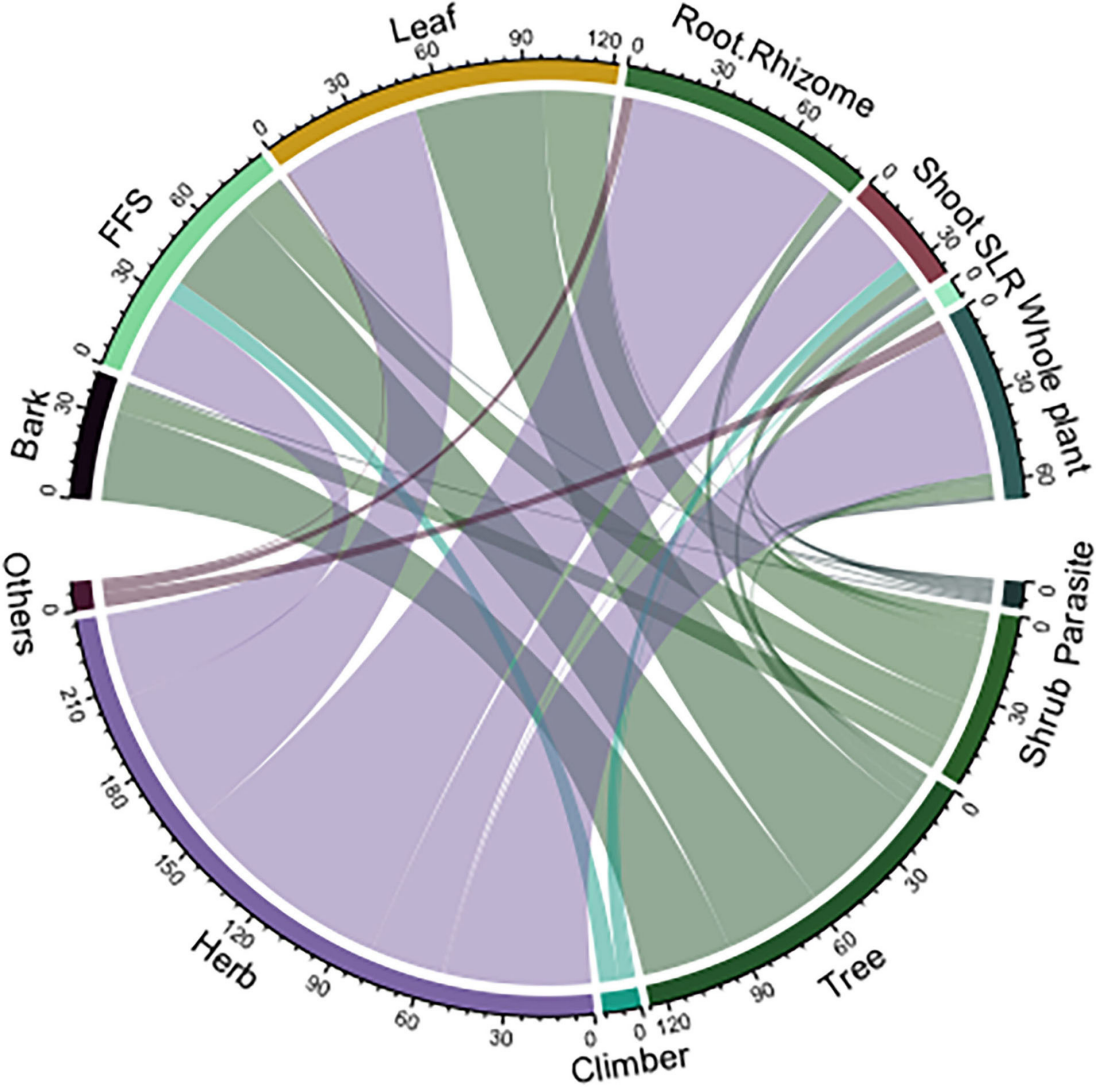
Chord diagram showing plant growth forms and parts used (see main text for abbreviations).

A chord diagram was used to see the relationship between plant growth forms and parts used (Figure 3). For this purpose, plant growth forms were differentiated into six forms, namely, climber, herb, shrub, tree, parasite, and others. Plant growth forms under the “Others” category include fern, fungus, and lichens. Likewise, plant parts were categorized into six forms for easier interpretation and presentation: whole plant, shoot, root, leaf, bark, flower/fruit/seed, and others. Flowers, fruits, seeds, and nectar were classified under one category and abbreviated as FFS, and stem and aerial parts were placed under “Shoot.” Roots, rhizome, tuber, and bulb were categorized and analyzed as “Root.” “Other” category includes Sap, Latex, and Resin (SLR). From the analysis of the diagram, we could assert that roots and leaves from herbs were the most frequently used parts whereas shrubs were less chosen for animal healthcare.

In terms of distribution of ethnoveterinary plant species, the highest number of species is reported from the mid-hills, followed by Tarai and Siwaliks, and mountains (Table 2). It is interesting to note that there is a correspondence between the relative abundance of different plant species that are present in a particular physiographic region and the number of ethnobotanical studies conducted in that region (Table 2). Mid-hills also hold the highest number of medicinal plants used by the humans (127). The possible reason for the use of medicinal plants both for livestock and humans in mid-hills could be attributed to the presence of a higher population of both livestock and humans and diverse ethnic groups (32, 33, 127). As most of these species have a wide distribution throughout Nepal, dissemination of information on the use of these species can promote their use for livestock management.

**Table 2:** Distribution of ethnoveterinary plant species in different physiographic regions of Nepal based on the literature.

**SN**	**Physiographic region**	**Number of studies**	**References**	Number of species^*^
1	Tarai and Siwaliks (60–1,500 m)	32	(27, 30, 39–68)	145
2	Mid-hills (1500–3,000 m)	60	(2–4, 18, 22, 26, 28, 29, 31, 69–118)	458
3	Mountains (3000–4,000 m)	12	(1, 20, 21, 23, 119–126)	70

* Species may be distributed in more than one physiographic region.

### Number of reported ethnoveterinary uses and livestock ailments treated

Of the total 393 species, 33 species were found to be most frequently cited in the literature, where *Cannabis sativa* had 24 citations followed by *Asparagus racemosus* (18), *Schima wallichii* (13), *Alstonia scholaris* (11), *Lindera neesiana* (11) and *Senna tora* (10). The species having a minimum of five citations are presented in Table 3.

**Table 3:** Most frequently cited ethnoveterinary plant species.

**Plant species**	**Family**	**Total number of citations**	**Ethnoveterinary uses**	**References**
*Cannabis sativa*	Cannabaceae	24	Anthelmintic, diarrhea, dysentery, cough, cold, veterinary problems, urinary problems, flatulence, stomachache, swollen stomach	(4, 20, 23, 26, 27, 30, 31, 40, 41, 45, 61, 62, 65, 71, 75, 80–82, 86, 105, 111, 113, 115, 118)
*Asparagus racemosus*	Asparagaceae	18	Stimulate lactation, stomack pain, colic, mastitis, bone problems, treat worms in hoof and stomach, remove placenta	(20, 26, 28, 31, 41, 43, 44, 62, 65, 68, 77, 78, 80, 81, 104, 108, 112, 116)
*Schima wallichii*	Theaceae	13	Taeniasis, stomach disorders, anthelmintic, diarrhea, cough	(4, 26, 28–31, 71, 86–89, 91, 100, 108)
*Alstonia scholaris*	Apocynaceae	11	Nutritious feed, sterility effect, diarrhea, dysentery, tonic, fever, increase lactation, for strength and vigor	(27, 29–31, 41, 42, 68–70, 74, 112)
*Lindera neesiana*	Lauraceae	11	Diarrhea, dysentery, antidote, remove placenta, tonic, indigestion, ectoparasites	(4, 26, 29, 31, 73, 80, 85, 106, 108, 109, 112)
*Senna tora*	Fabaceae	9	Treat falling of hairs, fever, anthelmintic, veterinary medicine	(29, 30, 39, 41, 52, 58, 82, 96, 102)
*Colebrookea oppositifolia*	Lamiaceae	9	Conjunctivitis, cataract, corneal opacity, anthelmintic, veterinary diseases, remove leech from nostril	(4, 28, 31, 39, 69, 75, 81, 89, 114)
*Achyranthes aspera*	Amaranthaceae	8	Veterinary medicine, accelerate explulsion of placenta, cure endoparasites, ease delivery, stimulates lactation	(26, 27, 30, 31, 39, 69, 104, 108)
*Boenninghausenia albiflora*	Rutaceae	7	Treat ectoparasites, wounds,	(4, 18, 26, 30, 31, 71, 114)
*Cuscuta reflexa*	Convolvulaceae	8	Pneumonia, asthma, cough, throat allergy, indigestion, stomach disorders, endoparasites, pain, fever, dysentery	(4, 26, 30, 48, 68, 93, 122, 128)
*Millettia extensa*	Fabaceae	8	Antiectoparasitic, veterinary medicine, scabies,	(28, 29, 31, 39, 51, 56, 67, 96)
*Lyonia ovalifolia*	Ericaceae	7	Skin disease, poisonous	(1, 2, 29, 48, 85, 114, 118)
*Oxalis corniculata*	Oxalidaceae	7	Earache, body swelling, veterinary medicine, boils, eye problems, muscular swelling	(4, 26, 27, 30, 31, 39, 70)
*Pyrus pashia*	Rosaceae	7	Lactation, eye problem including cataract, constipation	(23, 48, 88, 111, 117, 119, 126)
*Solena amplexicaulis*	Cucurbitaceae	7	Lactation, veterinary medicine, mastitis, intestinal worms	(22, 26, 28, 31, 39, 89, 116)
*Bombax ceiba*	Bombacaceae	6	Veterinary medicine, boils, constipation, dysentery, remove placenta, indigestion, dislocated bones, cut and wounds	(4, 26, 29, 30, 39, 64)
*Datura metal*	Solanaceae	6	Diarrhea, dysentery, fever, inflammation, wounds, joint swelling, induce sleep	(26, 27, 29, 30, 40, 114)
*Nicotiana tabacum*	Solanaceae	6	Skin disease, Antiectoparasitic, wounds, fever	(23, 29, 40, 75, 98, 106)
*Pogostemon benghalensis*	Lamiaceae	6	Dysentery, veterinary medicine, wound, cough, bronchitis	(26, 29–31, 39, 58)
*Prunus persica*	Rosaceae	6	Cut, wounds, bone dislocation, endoparasites	(26, 51, 63, 71, 112, 122)
*Stephania glandulifera*	Menispermaceae	6	Veterinary problems, tonic, stomach disorder, diarrhea	(28, 29, 31, 69, 70, 108)
*Acorus calamus*	Acoraceae	5	Repellent, indigestion, cough, fever	(22, 23, 26, 27, 40)
*Azadirachta indica*	Meliaceae	5	Anthelmintic, cuts, wounds	(26, 27, 30, 31, 82)
*Boehmeria virgata* var. *macrostachya*	Urticaceae	5	Diarrhea, dysentery, cuts, wounds	
*Clerodendrum infortunatum*	Lamiaceae	5	Veterinary medicine, remove lice, intestinal worms, stomach swelling, wounds	(27, 30, 39, 45, 46)
*Ficus religiosa*	Moraceae	5	Foot and mouth disease, rheumatism, urinary problem, treat burn, fever	(26, 27, 30, 40, 54)
*Rumex nepalensis*	Polygonaceae	5	Antidote, dislocated bones, diarrhea, tonic	(4, 22, 29, 31, 116)
*Tinospora cordifolia*	Menispermaceae	5	Cure sterility, increase lactation, appetite loss, cough, constipation, diarrhea	(26, 27, 31, 45, 91)
*Urtica dioica*	Urticaceae	5	Increase lactation, cure mastitis, urinary problems, sprain	(4, 26, 63, 76, 114)
*Viscum album*	Viscaceae	5	Dislocated bones, wounds, veterinary disease, treat swelling, boils	(20, 26, 31, 70, 80)
*Zingiber officinale*	Zingiberaceae	5	Foot and mouth disease, fever, diarrhea, mastitis, wounds, cough	(23, 26, 27, 29, 80)

The literature revealed that 213 plant species had a single ethnoveterinary use, while 180 species had multiple uses. Ninety-three species had more than two uses, where *Cannabis sativa* and *Cuscuta reflexa* had the highest number of reported uses (10), followed by *Bombax ceiba* (9).

Altogether, 34 ailments were treated with 393 species (Table 4). The highest number of remedies involving plants (111 species) were for the treatment of various gastrointestinal disorders followed by the species used to treat infections and infestations (65), genitourinary system disorders (60), pregnancy/lactation/puerperium disorders (60), and muscular-skeletal system disorders (59). Bartha et al. (8) also reported similar veterinary ailments treated by various plant species in Romania. Some of the most common infectious diseases prevailing in Nepal such as foot and mouth disease (FMD), and hemorrhagic septicemia (129) are also treated using a number of plant species in various ethnoveterinary practices. *Angiopteris evecta, Brucea javanica, Erythrina stricta, Ficus religiosa, Fragaria nubicola, Nyctanthes arbor-tristis, Prunus persica, Vigna radiata* and *Zingiber officinale* are used to treat FMD, whereas hemorrhagic septicemia is treated using species such as *Maesa macrophylla* and *Tridax procumbens*. With particular regard to the use of some plant species such as *Cannabis sativa* and *Cassia fistula* in the treatment of gastrointestinal disorders of livestock, ethnoveterinary practices in Nepal seem comparable to those reported in Pakistan (6). Conversely, the uses of *Allium sativum* and *Juglans regia*, for example, are different from those reported from Romania (8), indicating that cultural practices determine the ways in which plants are used (130). Herbal preparations were applied externally and internally based on the ailments.

**Table 4:** Number of plant species used to treat various livestock ailments/uses.

**Ailment category/uses**	Number of species^*^
Digestive System Disorders (Stomach disorders, constipation, diarrhea, dysentery, colic, indigestion, dyspepsia, bloat/tympany)	111
Infections/Infestations (Haemorrhagic septicaemia, anthelmintic, taeniasis, diphtheria, fever, foot and mouth diseases)	65
Pregnancy/Lactation/Puerperium Disorders (Abortion, delivery, galactogogue, increase lactation, agalactia)	60
Genitourinary System Disorders (Urinary disorders, diuretic, retention/explulsion of placenta, nipple infection/bovine haematuria, sterility in ox, cure sterility, aphrodisiac)	60
Muscular-Skeletal System Disorders (Sprain/pain/swelling, Facture/bone dislocation)	59
Injuries (Wounds)	58
General veterinary medicine	35
Antiectoparasitic (remove lice, ticks and other external parasites)	35
Skin/Subcutaneous Tissue Disorders (Skin problems, skin ring, burns and boils, hoof infection, falling of hair)	35
Nutritional disorders (Tonic, appetizer/anorexia)	32
Respiratory System Disorders (Asthma, cough, pneumonia)	31
Sensory System Disorders (Cataract/conjunctivitis/eye problems, earache)	24
Antipoisioning (used as antidote and for intoxication)	16

* A species may be used to treat more than one disorder.

Livestock species in which ethnoveterinary uses of plant species were practiced are not specified in the majority of the literature. The majority of them had broad indications such as “used in veterinary medicine,” (39, 113) “given to animals,” (110) and “remove lice from the body of animals” (26). Only a few literature have specifically indicated the livestock species such as “fed to cattle as anthelmintic medicine,” (71) “antiseptic on wounds in cattle,” (2) “applied to get rid of lice and ectoparasites of the sheep,” (31) “given to cows to increase milk production,” (77) “given to sterile female buffalo/cow,” (97) and “given to yaks and sheep to relieve from fever” (4).

Twenty-four species of the plants are also reported to be poisonous to cattle. The most cited poisonous plant species is *Lyonia ovalifolia* (1, 29, 48, 85, 114, 118). The knowledge regarding various toxic and poisonous species is considered a prerequisite for safe grazing as grazing on such species could be fatal resulting in economic loss and thus this knowledge holds value for healthy livestock farming (35). Among toxic and poisonous species, some are used externally to treat wounds and skin diseases (*Boenninghausenia albiflora, Bupleurum candollei, Crotalaria spectabilis, Lyonia ovalifolia*, and *Pieris formosa)* and as an antiectoparasitic (*Boenninghausenia albiflora, Prunus armeniaca*); while leaves are poisonous, the bark juice of *Osyris wightiana* is given in indigestion.

## Conclusions

The present review revealed that Nepal has a rich diversity of ethnoveterinary plants. This study further shows that traditional herbal medicine is playing a significant role in meeting the livestock healthcare needs of Nepali farmers and hence is a sustainable practice. Some of the plants reported in the literature are widely used and also abundantly available in the wild. Further studies on phytochemical and pharmacological profiles, including toxicological and clinical studies of interesting ethnoveterinary plants are necessary to contribute to modern veterinary health care choices based on these traditional herbal remedies. It is equally important to safeguard the traditional knowledge and local flora by raising awareness among the local people about the importance of their knowledge and plants. If further research and development are possible by using traditional knowledge leading to drug discovery, the access and benefit sharing process and laws under the Nagoya Protocol should be applied to ensure the rights and responsibilities of users and providers of genetic resources and associated traditional knowledge (131).

## Author contributions

YU, RK, and RP designed the study, reviewed the literature, analyzed the data, and refined the drafts. SK and RP cross-checked the data and analyses and reviewed the drafts. All authors proofread the manuscript.

## Funding

Partial funding for article processing charge was received from the University Grant Commission, Nepal and Nepal Academy of Science and Technology, Lalitpur. Authors are thankful to these funding organizations.

## Conflict of interest

The authors declare that the research was conducted in the absence of any commercial or financial relationships that could be construed as a potential conflict of interest.

## Publisher's note

All claims expressed in this article are solely those of the authors and do not necessarily represent those of their affiliated organizations, or those of the publisher, the editors and the reviewers. Any product that may be evaluated in this article, or claim that may be made by its manufacturer, is not guaranteed or endorsed by the publisher.
