# Supplementary material for: Ethnoveterinary use of plants and its implication for sustainable livestock management in Nepal
Source: Front Vet Sci. 2022 Sep 9;9:930533. doi: 10.3389/fvets.2022.930533 (PMC9500146; doi:10.3389/fvets.2022.930533)
Supplement: Supplementary file 1 [file Table_1.pdf]

Supplementary File: Ethnoveterinary uses of plants in Nepal

SN	Scientific name (synonyms with asterisk)	Habit	Family	Local name	Parts used	Use
1	*Abelmoschus esculentus* (L.) Moench	Herb	Malvaceae	Bhindi (Nepali)	Roots	Crushed and fed to cure urinary related problems (26), juice is given to relieve blocked urination (29).
2	*Abelmoschus manihot* (L.) Medik.	Herb	Malvaceae	Som raja (Nepali)	Roots	Paste used to treat bone dislocation (29).
3	*Abrus precatorius* L.	Climber	Fabaceae		Seeds	Fed for placental retention with quicker parturition (27).
4	*Achyranthes aspera* L.	Herb	Amaranthaceae	Apamarga, Datiun, Chir-chiri, Ulata kara (Nepali)	Whole plant, roots	Plant used as veterinary medicine (39). Whole plant and roots used to accelerate the expulsion of placenta (72,109); used for placental retention with quicker parturition (27). Whole plant is fed as a feed to cure endoparasite (26). Root piece is tied around the neck of the cattle to make it easy in delivery (30,31). Root juice stimulates lactation in buffaloes (104).
5	*Acmella calva* (DC.) R. K. Jansen<br><br>******Spilanthes calva* DC.	Herb	Asteraceae		Whole plant	Plant paste is applied as an antidote to snake bite (31).
6	*Aconitum gammiei* Stapf	Herb	Ranunculaceae		Rhizome	Used as antipoisoing (122).
7	*Aconitum heterophyllum* Wall. ex Royle	Herb	Ranunculaceae	Atish, bish (Nepali)	Roots/tuber	Whole plant is poisonous to cattle (119).

8	*Acorus calamus* L.	Herb	Acoraceae	Bojho (Nepali); Seda, Sete (Tamang); Sutak, Ugragandha (Sanskrit); Katara (Tharu); Charila, Chota (Gurung); Chhurcha, Syueda (Sherpa); Shu-dag (Tibetan); Bojha (Satar); Bach (Hindi); Sweet flag (English)	Rootstock, rhizome	Paste used to remove lice from the body of animals (22,26). Rhizome used in indigestion (40,27), treat external parasite and cough (23). Paste of this plant with *Zingiber officinale* and *Piper nigrum* is given in fever (27).
9	*Adiantum venustum* D. Don	Fern	Pteridaceae	Sinke (Nepali), Bhasipsip (Tamang)	Leaves, rhizome	Juice used externally in cuts and septic wounds particularly for yak and nak (4).
10	*Adina cordifolia* (Roxb.) Brandis	Tree	Rubiaceae	Karma (Nepali)	Leaves	Young leaves are poisonous to cattle (70).
11	*Aegle marmelos* (L.) Corrêa	Tree	Rutaceae	Bel (Nepali)	Leaves, fruits	Fruit is crushed and the paste given to treat fever (26). Fresh leaves are given to the cattles to control diarrhea and intestinal worms. Leaf decoction is used to treat wounds (30).
12	*Aeschynomene indica* L.	Herb	Fabaceae		Whole plant	Used as veterinary medicine (39).
13	*Aesculus indica* (Colebr. ex Cambess.) Hook.	Tree	Sapindaceae	Panger, Karu (Nepali)	Seeds, fruits, Leaves	Eases urinaton of livestock (34). Cotyledons are fed to cattle as an anthelmintic medicine (71). Fresh leaves are fed to animals as anthelmintic (28).

						Pounded seeds given to treat dysentery (29).
14	*Agave cantala* var. *cantala*<br><br>**Agave cantula* Roxb.	Shrub	Agavaceae	Ketuki, Rambans, Hattibar (Nepali)	Leaves	Paste used in worms of livestock (120). Juice extracted from boiled leaf is given orally to cure fever, grounded to obtain juice and fed for any urinary related problem (26). Juice rubbed locally to treat boils (29).
15	*Albizia chinensis* (Osbeck) Merr.	Tree	Fabaceae		Leaves and Shoots	Poisonous to cattle (29).
16	*Albizia julibrissin* Durazz.	Tree	Fabaceae	Siris (Nepali)	Whole plant	Poisonous to cattle (72).
17	*Albizia procera* (Roxb.) Benth.	Tree	Fabaceae	Jukret (Nepali)	Bark	Dried bark is crushed to make powder, and drenched with water to cure bone related problems (26).
18	*Ageratum conyzoides* L.	Herb	Asteraceae	Bhera jhar (Nepali)	Leaves	Paste used to treat dislocated bones (29).
19	*Alocasia indica* (Lour.) Spach	Herb	Araceae		Young stem	Roasted and fed as aphrodisiac (27).
20	*Aloe vera* (L.) Burm.f.	Herb	Asphodelaceae	Ghyu kumari (Nepali)	Leaves, Jel	Jelly portion of leaf is rubbed in burn area and cure mastitis (26). Leaves used in veterinary medicine (113).
21	*Allium cepa* L.	Herb	Amaryllidaceae	Pyaj (Nepali)	Bulbs	Used in indigestion/anorexia (23) and constipation (27).
22	*Allium sativum* L.	Herb	Amaryllidaceae	Lasun (Nepali), No (Magar), Nasun (Tharu),	Bulbs	Grounded and mixed with dried human placenta (Nâbi) and given to cattle to cure poison (73). Used in indigestion/anorexia (23). Paste with *Semecarpus anacardium* is used to treat tail cut (27). Mixed with

				Garlic (English), Rosun (Sanskrit)		*Mentha arvensis* and given to animal to cure diarrhea (26).
23	*Alstonia scholaris* (L) R. Br.	Tree	Apocynaceae	Chhatiwan (Nepali)	Bark, Seeds, Sap	Bark powder mixed with some grain flour is considered a nutritious feed for cattle (70). Bark powder is a feed suppliment to increase sterility to ox (74). Mixture prepared with other species is given to the animal in diarrhea and dysentery (41). Bark powder is given as tonic, cause sterility effect on female cattle (69). Bark used as tonic (42), paste given in fever (27). Bark paste mixed with flour is given to eat cattle as tonic to increase weight (112). Juice with other ingredients is used to treat diarrhea (29). Bark decoction is given to cattles to reduce fever (30). Juice is given in diarrhea and dysentery, used to increase lactation (68). Seed powder mixed with corn flour is given to cattle for strength and vigour (31).
24	*Amaranthus spinosus* L.	Herb	Amaranthaceae		Roots	Juice given to animals in urine trouble (100).
25	*Amaranthus viridis* L.	Herb	Amaranthaceae	Lude (Nepali), Thadiya generi (Bantar)	Aerial parts	Given to increase lactation (40,27).
26	*Ampelocissus divaricata* (Wall. ex M.A.Lawson) Planch.	Climber	Vitaceae	Purreni (Nepali)	Sap	One end of fresh-cut stem is brought near to eye and watery like substance oozing out from stem is let go into eye by blowing air (26).

27	*Anaphalis triplinervis* (Sims) C. B. Clarke	Herb	Asteraceae	Ekle ghans (Nepali)	Flower	Paste is applied as an antiseptic on wounds in cattle (2).
28	*Anemonastrum obtusilobum* subsp. *obtusilobum*<br>**Anemone obtusiloba* D. Don	Herb	Ranunculaceae	Maulomuro (Nepali)	Roots	Used to treat external parasite (23).
29	*Anemonastrum elongatum* (D. Don) Holub<br>**Anemone elongata* D. Don	Herb	Ranunculaceae	Dhanero (Nepali)	Whole plant	Plant extract is applied to get rid of lice and ectoparasite of the sheep (31).
30	*Angiopteris evecta* (G. Frost.) Hoffm	Fern	Marattiaceae	Gaikhure uneu (Nepali), Bigtagrab (Lepcha)	Rhizome	Paste is used in foot and mouth disease for cattle (112).
31	*Anisomeles indica* (L.) Kuntze	Herb	Lamiaceae	Rato charpate (Nepali), Bar (Bantar)	Leaves	Leaves used in constipation and tiredness (43).
32	*Annona squamosa* L.	Tree	Annonaceae	Sarifa, Sita phal (Nepali)	Leaves	Used to treat germs and worms of domestic animals (40). Decoction is used in the treatment of wounds. Paste is applied to the animals to remove lice and other external parasitic insects (30).
33	*Arctium lappa* L.	Herb	Asteraceae	Kurya (Nepali)	Roots	Decoction is given to cattle to kill intestinal worms (3).
34	*Argemone mexicana* L.	Herb	Papaveraceae		Seeds, Roots	Seed oil used to treat eye complaint (27). Grinded root is used to cure stomach disorder of pigs (101).

35	*Arisaema tortuosum* (Wall.) Schott.	Herb	Araceae		Seeds	Used to treat diarrhea and dysentery in goat and sheep (29).
36	*Artemisia carvifolia* Buch.-Ham. ex Roxb.	Herb	Asteraceae	Titepati (Nepali)	Whole plant	Plant is given to horses during the winter season to keep the body warm (31).
37	*Artemisia indica* Willd.	Herb	Asteraceae	Titepati (Nepali), Mugwort (English)	Leaves	Fresh leaf juice is applied to bleeding wounds and cuts as a haemostatic and an antiseptic in cattle (2). Leaves used to treat expernal parasite (23,26). This plant with *Azadirachta indica* and *Melia azedarach* are taken and cut into pieces, mixed with cornflour, cooked, and fed to cure endoparasite (26). Leaf and camphor are crushed to make a paste and applied in a wounded area twice a day (26).
38	*Artocarpus heterophyllus* Lam.	Tree	Moraceae	Katahar (Nepali)	Leaves, Fruits	Leaves is directly fed to cattle to control cough (75). The paste of inner part of the fruit is applied to treat mastitis (28). Ripen fruit is fed directly, and the fruit core is applied in teats to cure mastitis (26). Fresh leaves are fed to goats to check diarrhea (30).
39	*Asparagus filicinus* Buch.-Ham. ex D.Don	Herb	Asparagaceae	Kurilo (Nepali)	Tender parts, Roots	Tender parts are given to female cattle in case of lactation problems (76). Root juice is given for more milk production (100).
40	*Asparagus officinalis* L.	Herb	Asparagaceae	Santawar, Kurla	Roots	Used to increase lactations of cattles. Powder is given as supplementary diet to cattles (30).

41	*Asparagus racemosus* Willd.	Herb	Asparagaceae	Kurilo, Satawari, Jhirjhire kanda (Nepali); Kurila (Raji); Sunga, Komo (Tamang); Kurla (Tharu), Satmuli (Sanskrit.); Pustu, Lhodu (Gurung)	Roots	Tuberous roots with other ingredients are fed to the nursing livestock (77). Powder is given as a tonic to a lactating postpartum animal (41,108) and to treat milching disorder (108). Crushed and given to lactating livestock (20,43,80,116). Juice is given as remedy for stomach pain, colic in cattle. Paste is mixed with cattle feed to increase lactation (27,28,81); administered as an invigorating tonic to lactating livestock; a branch is hung in the shed to help sick livestock (44) and also to treat mastitis (28). Crushed and mixed with cattle feed for any bone related problem and mastitis (26). Juice is mixed with corn flour and given to the buffaloes to stimulate milk production (104). Juice is given in case of any worms in hoof or in the stomach, helpful to remove placenta (62). Tubers are cooked and given to cattle to promote lactation (65,68,112). Fed to treat milching disorder of cattle (31).
42	*Auricularia nigricans* (Sw.) Birkebak, Looney & Sánchez-García<br><br>**Auricularia polytricha* (Mont.) Sacc.	Fungus	Auriculariaceae		Whole plant	Given in dyspepsia (80).
43	*Avena sativa* L.	Herb	Poaceae	Jai ghans (Nepali)	Leaves	Grass is fed to increase lactation (28).

44	*Azadirachta indica* A. Juss.	Tree	Meliaceae	Neem (Nepali), Margosa tree (English)	Leaves, Bark	Fresh leaves is made into paste and prescribed to mature cattle as an anthelmintic (82); fed to treat intestinal worms (27). Paste is made by crushing leaf and applied to cure ectoparasite, and fed to cure wound; this plant with *Artemisia indica* and *Melia azedarach* are taken to prepare formulations and given to cure endoparasites (26). Young leaves are given to cattles to check intestinal worms. Decoction of bark is used to wash cuts and wounds (30). Paste is applied to heal wounds (31).
45	*Bauhinia malabarica* Roxb.	Tree	Fabaceae	Tanki (Nepali)	Flower	Used to treat wounds in neck (80).
46	*Bauhinia purpurea* L.	Tree	Fabaceae	Koiralo (Nepali)	Leaves	Fed to enhance lactation (26).
47	*Bauhinia vahlii* Wight & Arn.<br>**Phanera vahlii* (Wight & Arn.) Benth.	Climber	Fabaceae	Bharla (Nepali)	Bark	Dried bark is grounded to make powder and fed with honey to treat joint-related problem (26).
48	*Bauhinia variegata* L.	Tree	Fabaceae	Koiralo (Nepali)	Leaves	Given to cows to increase milk production (77).
49	*Begonia picta* Sm.	Herb	Begoniaceae	Makar kanchi (Nepali)	Whole plant, Roots, Stem	Infusion of the root is applied to treat conjunctivitis of buffalos (26). Used to treat diarrhea and dyspepsia (105). Plant is fed to sterile animals to conceive (107). Plant is applied to the animals to wake the barren cattle fertile (31).

50	*Benincasa pruriens* f. *hispida* (Thunb.) De Wild. & Duyfjes<br><br>**Benincasa hispida* (Thunb.) Cogn.	Herb	Cucurbitaceae	Kubhindo (Nepali)	Fruits	Used in abortion (23).
51	*Berberis aristata* DC.	Shrub	Berberidaceae	Chutro (Nepali); Daruharidra, Dwari (Sanskrit); Komme(Gurung) ; Cautara (Magar); Trikhula (Raji); Barberry (English)	Fruits, Roots, Bark	Fruits are fed to animals as pain reliever (83), decoction of root is taken to kill intestinal worms of cattle (81,116). Squeezed bark is fed to treat stomach troubles (107).
52	*Bergenia ciliata* (Haw.) Sternb.	Herb	Saxifragaceae	Silphode (Nepali)	Roots, Rhizomes, Fruits	Roots and fruits are grounded with other ingredients and fed to the post-delivery livestock to increase lactation and keep livestock healthy (77). Roots used in diarrhea and dysentery (23). Rhizome paste used to treat bone facture (118).
53	*Betula utilis* D. Don	Tree	Betulaceae	Bhojpatra (Nepali)	Bark	Used to treat sprain/pain (23).
54	*Bistorta milletii* Lév.	Herb	Polygonaceae	Myakhuri (Nepali)	Roots, Fresh plant	Roots used to treat diarrhea and dysentery (23). Fresh plant is given as nutritious food and to enhance lactation to nak (4).
55	*Bistorta vacciniifolia* (Wall. ex Meisn.) Greene	Herb	Polygonaceae	Phulunge jhar (Nepali); Tolowang (Tamang)	Roots	Juice is given orally to relief the fever (4).

56	*Boehmeria virgata* var. *macrostachya* (Wight) Friis & Wilmot-Dear<br><br>**Boehmeria platyphylla* D. Don	Shrub	Urticaceae	Gargalo (Nepali)	Roots, Whole plant	Root paste is given to cattle to treat diarrhea (70). Decoction of the plant is given to livestock for diarrhea and dysentery; root paste is used to treat cattle wounds and cuts (84, 85). Plant decoction is given to cattle to check diarrhea (30, 31).
57	*Boenninghausenia albiflora* (Hook.) Rchb. ex Meisn.	Herb	Rutaceae	Uruse jhar, Dampate (Nepali); Urmen (Tamang)	Whole plant, Leaves	Plant is poisonous to animal (71). Juice of plant is spread to get rid of bugs; it is also applied on the body to remove lice or other ectoparasites on the body of domestic animals (18). Plant powder is applied all over the body externally to remove the parasitic insect (4). Fresh twigs and leaves are grounded with camphor and applied to the wound (26). Fresh plant or smoke of dreid plant is used in repelling fleas of poultry (101). Leaf juice of plant is applied over body to remove lice and other external parasite and insects (26,30,31).
58	*Bombax ceiba* L.	Tree	Bombacaceae	Simal (Nepali); Kakdhong (Tamang)	Bark, Flowers, Seeds, Whole plant	Plant used as veterinary medicine (39). Bark extract is applied for the boils (4). Flower is crushed and fed to cure constipation; powder obtained from bark is mixed with powder of pepper and ginger, and then given orally to cure dysentery; raw bark is crushed to obtain juice and given to remove the placenta; seeds are grounded with mustard oil, and then applied to treat the wounds (26,49). Seed paste used to treat indigestion and dislocated bones (29). Bark decoction is

						given to cattle to check dysentery, reduce inflamations; bark paste is applied locally to get relief from cut and wounds (30).
59	*Brassica napas* L.	Herb	Brassicaceae		Seeds	Warmed oil used to treat chronic cough (27).
60	*Brassica nigra* (L.) W.D.J.Koch	Herb	Brassicaceae	Tori (Nepali)	Seeds	Mustard cake is mixed with maize flour, and fed to increase milk production, cake is applied to cure scabies (26).
61	*Brassica rapa* L.<br>**Brassica campestries* L. var. *sarson* Prain	Herb	Brassicaceae	Salagam (Nepali)	Seeds	Seed oil cake is useful for cattle food (84).
62	*Brassica* sp.	Herb	Brassicaceae		Roots	Used to treat bloat/tympani (23).
63	*Bridelia retusa* (L.) A. Juss.	Tree	Phyllanthaceae	Gayo (Nepali)	Young leaves	Young leaves are poisonous to cattle (86).
64	*Brucea javanica* (L.) Merr.<br>**Rhus javanica* L.	Tree	Anacardiaceae	Bhaki amilo (Nepali); Bhakmilo (Tharu), Muruk (Magar)	Fruits	Decoction is given to animals in case of foot and mouth diseases (84,87). Ripe fruits are used as appetizer for livestock (88), given to treat diarrhea and dysentery (26).
65	*Bupleurum candollei* Wall.	Herb	Apiaceae	Tsa Duk (Sherpa)	Leaves, Roots	Leaves are poisonous to cattle (1,25,29); root juice is dropped in the wound to kill germs and heal quickly (29).
66	*Caesalpinia decapetala* (Roth.) Alston	Shrub	Fabaceae	Ulte kanda (Nepali)	Roots	Juice is applied to treat sprain and muscular swelling of animals (71).
67	*Caesulia axillaris* Roxb.	Herb	Asteraceae	Phalewa, Thukaha (Danuwar)	Whole plant	Juice given to domestic animal as tonic (89).

68	*Cajanus scarabaeoides* (L.) Thouars<br><br>**Atylosia scarabaeoides* (L.) Benth.	Herb	Fabaceae	Bhatmase ghans (Nepali)	Fresh plant	Fresh plant is given to the cattlle in diarrhea (4). Plant juice is given to treat diarrhea (29).
69	*Callicarpa macrophylla* Vahl	Tree	Lamiaceae	Gunelo (Nepali)	Leaves	Used as veterinary medicine (39).
70	*Calotropis gigantea* (L.) W. T. Aiton	Shrub	Apocynaceae	Aank (Nepali)	Leaves, Roots	Lightly crushed leaves are warmed on fire and kept on swelling joint (26). Juice used to treat diarrhea with other ingredients; root paste with other ingredients is used to treat wounds on shoulder of an ox or a buffalo (29). Leaves caused diarrhea if the fodder is given in winter (31).
71	*Calotropis procera* (Aiton) Aiton fil.	Shrub	Apocynaceae	Akon	Latex	Latex drop is given orally to reduce stomachache of domestic animals; latex mixed with honey is applied locally to reduce inflamations on the body (30).
72	*Campylotropis speciosa* (Schindl.) Schindl.	Shrub	Fabaceae	Sakino (Nepali), Mena (Tamang)	Leaves	Paste is applied over the dislocated bones to hasten up the process of healing particularly for the cows (4).

73	*Cannabis sativa* L.	Herb	Cannabaceae	Ganja, bhango (Nepali); Wang/Sima (Tamang), Gagima, Kantsya (Gurung); Bhanga (Raji); Hemp (English)	Leaves, seeds, inflorescen ce	Seed decoction is given to mature cattle along with other cooked foods for its alleged anthelmintic action (82). Plant juice is given to cattle to cure diarrhoea (45). Decoction of leaves is fed to cure diarrhea (31,61,75). Power of dried leaf is fed to animals in case of diarrhea (62), cough and cold (71). Seed decoction with other ingredients is given to cattle in diarrhea (41). Leaf powder is mixed with cattle feed to treat veterinary problems (20). Leaves used to treat diarrhea and cold (23,113). Fresh leaves and inflorescence are fed to treat the diarrhea (4,49,80,81,86,105). Paste from flowering tops is given to treat diarrhea (27). Seeds given to livestock to treat dysentery (115). The mixture of leaf and seed is grounded then drenched with water to cure diarrhea, fever, and urinary related problems (26). Leaves are fed to animals to treat diarrhea (111). Infusion of young leaves and inflorescens with fruits of *Datura metel* is given to cattle in flatulence and stomachache (65). The twig is fed to animals to check diarrhea (30). Roasted seeds given to cattle to treat diarrhea and to get relief from swollen stomach (118).
74	*Capsicum annuum* L.	Herb	Solanaceae	Khursani (Nepali)	Fruits	Directly given to the animal to treat urinary related problem (26). Ripe fruits are used to cure chronic cough disease of the domestic cattle (30).

75	*Capsicum frutescens* L.	Herb	Solanaceae	Jire khursani (Nepali)	Leaves	Paste with other ingredients is used to treat mumps (29).
76	*Cardiospermum halicacabum* L**.**	Climber	Sapindaceae	Kesh lahara (Nepali)	Roots	Paste used to remove lice or any other ectoparasites (29).
77	*Carica papaya* L.	Tree	Caricaceae	Mewa (Nepali)	Seeds	Matured dry seeds are crushed to make powder, and given orally to cattle against internal parasites (26).
78	*Carissa carandas* L.	Shrub	Apocynaceae		Roots	Decoction is given in case of blood dysentery of cattle (31).
79	*Carthamus tinctorius* L.	Herb	Asteraceae	Kusum (Nepal)	Seeds	Oil is applied over the wound to 'Khorahiya' cure foot and rot disease (30).
80	*Cassia fistula* L.	Tree	Fabaceae	Amaltas, Rajbrikshya (Nepali)	Fruits, Seeds Bark, Leaves, Roots	Paste of seeds applied around the umbilicus (belly button) as a diuretic (41). Grinded seeds with other ingredents is given to cattle for treating urinary disorder (120). Dried seeds and fruits are grounded to make powder, and fed to cure urinary problem (26).
81	*Cassiope fastigiata* (Wall.) D. Don	Shrub	Ericaceae	Phursan (N.), Nyamlu (Tam.)	Aerial parts	Powder is applied all over the swellings of the limb which are believed to be due to ghost; plants are also given to cattles for lactation (4).
82	*Cedrus deodara* (Lamb.) G. Don	Tree	Pinaceae	Devadar (Nepali)	Resin	Resin is used to treat external parasite (23).
83	*Centella asiatica* (L.) Urb.	Herb	Apiaceae	Ghodtapre (Nepali)	Whole plant	Plant used as veterinary medicine (39). Fresh plants are fed to the horses in case of the urinary problems (4,31). Leaves are roasted, cooled and given in diarrhea (27).

						Whole plant is fed to animals to treat urinary disorders (26,28).
84	*Chenopodium album* L.	Herb	Amaranthaceae	Bethe (Nepali)	Whole plant	Crushed and obtained juice is given orally to treat dysentery (26).
85	*Chromolaena odorata* (L.) R. King & H. Rob.	Shrub	Asteraceae	Seto Banmara (Nepali)	Leaves	Decoctionis applied to stop bleeding as well as skin ring of cattle (75).
86	*Cirsium verutum* (D.Don) Spreng.	Herb	Asteraceae	Thakal kanda (Nepali), Pongjugale (Tamang)	Roots	Juice is given to yaks and naks in cases of constipation and urinary problem (4).
87	*Cissampelos arenicola* M. Nee & R. Ortiz	Climber	Menispermaceae		Aerial parts	Used to treat bleeding (115).
88	*Cissampelos pareira* L. var. *hirsuta* (Buch.-Ham. ex DC.) Forman	Climber	Menispermaceae	Musya belo (Nepali)	Roots	Juice is dropped on the wounds of animals to dispel any germs and worms (90); used to treat urinary problems (79) and also given to treat tapeworm (80).
89	*Cissus quadrangularis* L.	Climber	Vitaceae	Hadjor (Nepali)	Stem	Stem paste is applied to cure dislocated bone of animals (30).
90	*Cissus repens* Lam.	Climber	Vitaceae	Charchare laharo (Nepali)	Stem	Stem is gently crushed to obtain juice and applied to any eye problem (26).
91	*Citrus medica* L.	Tree	Rutaceae	Bimiro, Mahulunga, Chuk (Nepali)	Fruits	Juice given orally to cattle against ascaris (91). Juice obtained by pounding root and given orally to treat endoparasite (26). Juice is used as anti-poison remedy (29).

92	*Citrus grandis* (L.) Osbeck<br><br>**Citrus maxima* Merr.	Tree	Rutaceae		Leaves	Leaves are crushed and mixed with cornflour to cure endoparasite; juice obtained is mixed with salt and applied to eye problem (26).
93	*Citrus limon* (L.) Burm. f.	Tree	Rutaceae	Nibuwa (Nepali)	Fruits	Juice is fed to treat stomachache (28). Lemon juice is mixed with egg. If head smut infected corn is available then its powder is mixed and given to animal to cure fever; juice is rubbed to swelling area (26).
94	*Clausena excavata* Burm. fil.	Shrub	Rutaceae	Bhanbhaniya (Danuwar)	Whole plant	Used to remove ectoparasites of poultry (46).
95	*Clausena pentaphylla* Lam. ex DC.	Tree	Rutaceae		Whole plant	Plant used as veterinary medicine (39).
96	*Clematis buchananiana* DC.	Climber	Ranunculaceae	Junge lahara (Nepali), Nasarpelandu (Tamang)	Leaves	Pounded and applied all over the burns and fed to the yaks for the inflammation during the burn (4).
97	*Clematis graveolens* Lindl.	Climber	Ranunculaceae	Nasar lahara (Nepali)	Whole plant	Paste applied to treat wound on the shoulder of an ox or a buffalo, caused by yoke (29). Given to horse during winter to keep body warm (31).
98	*Clerodendrum infortunatum* L.<br><br>**Clerodendrum viscosum* Vent.	Shrub	Lamiaceae	Bhanthi (Danuwar), Bhant (Nepali)	Leaves, Young shoots	Plant used as veterinary medicine (39). Leaf juice is applied on cattle body to remove lice (45,46). Leaves juice is fed to treat intestinal worms (27). Decoction of young shoots is given to animals to get relief from stomach swelling caused by indigestion. Leaf juice is applied over the

						body and wounds of animals as an insecticide (30).
99	*Codonopsis viridis* Wall.	Climber	Campanulaceae		Aerial parts	Used to treat constipation in cattle (121).
100	*Coelogyne cristata* Lindl.	Herb	Orchidaceae	Chandi gava (Nepali)	Bulb	Juice applied to skin boils and wounded hooves of cattle (92).
101	*Colebrookea oppositifolia* Sm.	Shrub	Lamiaceae	Dhurseli (Nepali)	Leaves	Juice is put into the eye to treat conjunctivitis (28,31,81,89), cataract (101), corneal opacity (28,69) and as an anthelmintic in cattles (4,31). Juice of young bud is applied to remove leech from nose of cattle (75). Plant used as veterinary medicine (39).
102	*Colocasia esculenta* (L.) Schott	Herb	Araceae	Karkalo (Nepali)	Leaves, Stem	Small pieces are made by cutting them and given to animal by cooking with cornflour to cure endoparasite and diarrhea (26).
103	*Commelina benghalensis* L.	Herb	Commelinaceae	Kane jhar, Kana gujguj (Nepali)	Whole plant, Leaves	Used as an antiseptic (91). Paste obtained from leaf is made and applied locally on fresh wounds in animals as antiseptic (26). Juice is applied locally on fresh cuts in animals as an insecticide (30).
104	*Corchorus capsularis* L.	Shrub	Malvaceae	Jute Plant (Nepali)	Stem	Stem is burned, and obtained ash is mixed with mustard, then fed to treat diarrhea (26).
105	*Coriandrum sativum* L.	Herb	Apiaceae	Dhaniya (Nepali)	Aerial parts	Given for better milk production (80).
106	*Crateva unilocularis* Buch.-Ham	Tree	Capparaceae	Simlikan (Nepali)	Leaves, Stem	Directly fed to cure endoparasite (26).

107	*Crinum amoenum* Ker Gawl. ex Roxb.	Herb	Amaryllidaceae		Rhizomes	Given in case of dysentery of cattle (31).
108	*Crotalaria spectabilis* Roth.	Herb	Fabaceae	Bhuban jhar (Nepali)	Leaves and shoots	Tender leaves and shoots are considered poisonous to cattle; paste of tender leaves is applied to treat wounds and with other ingredients it is used to treat muscular swelling (29).
109	*Croton caudatus* Geiseler	Shrub	Euphorbiaceae		Leaves	Plant is poisonous (101).
110	*Cryptolepis buchananii* Schult. ex Roem. & Schult.	Climber	Apocynaceae	Seto dhudiya (Tharu), Dudh Lati	Leaves, whole plant	Leaves fed to animal as galactagogue (47). The plant is hung around the neck of wheezing livestock (44); tied around the neck of the cattles to get relief from swelling (30).
111	*Cucumis maderaspatanus* L.<br>**Mukia maderaspatana* (L.) Roem.	Climber	Cucurbitaceae	Ladbhadi (Bantar), Sunkeshre laharo (Nepali)	Seeds, Leaves, Fruits	Seed paste is applied to treat scabies of animals (86). Squeezed fruit is applied to treat scabies in animals (87). Leaves used in constipation and tiredness (40).
112	*Cucumis melo* L.	Climber	Cucurbitaceae	Kharbuja (Nepali)	Seeds	Used in abortion (23).
113	*Cucumis sativus* L.	Climber	Cucurbitaceae	Kakro (Nepali)	Whole plant, Seeds	Whole plant is boiled with food and given to cattle for the expulsion of placenta (3). Used to treat blood in urine with other plants (27). Dried seeds are crushed to make powder and fed by drenching with water to cure urinary problems (26).

114	*Cucurbita maxima* Duchesne	Climber	Cucurbitaceae	Pharsi (Nepali)	Whole plant, Fruits	Given to cattle for expulsion of remained placenta and also indigestion in cattle (3). Ripen yellow pumpkin is cooked with cornflour and feed to cure mastitis (26).
115	*Cucurbita pepo* L.	Climber	Cucurbitaceae	Pharsi, Kadu (Nepali)	Fruits	Cooked and given to cattle for expulsion of remained placenta, laxative (3).
116	*Curculigo orchioides* Gaertn.	Herb	Hypoxidaceae	Kalo musali (Nepali)	Roots, Leaves	Roots used as aphrodisiac for cattle (19). Tender leaf juice is used to treat eye problems (29).
117	*Curcuma angustifolia* Roxb.	Herb	Zingiberaceae	Besar (Nepali)	Rhizomes	Decoction is given to domestic animals to treat constipation (116).
118	*Curcuma aromatica* Salisb.	Herb	Zingiberaceae	Kalo haledo, Kachur, Banhaldi, Besar (Nepali); Hardi (Magar); Turmeric (English)	Rhizomes	Used to cure broken horn in cattle (91), used as buti, food poisoning “Nas-Kapat" (69).
119	*Curcuma longa* L.	Herb	Zingiberaceae	Besar (Nepali)	Rhizomes	Used to treat external wounds (23). Dried rhizome is crushed and mixed with mustard oil. Then, applied in burn area, wound and to treat ectoparasite; little powder is blown to the eye to treat eye-related problem (26).

120	*Cuscuta reflexa* Roxb.	Parasite	Convolvulaceae	Akashlati, Amarlati, Nagbeli (Nepali)	Shoots, Whole plant	Shoot is used for pneumonia and cough of cattle (17). Whole plant used to prepare medicine to treat livestock with cough and throat allergy (93). Plant juice is given with crushed garlic to cattle in case of indigestion (48) and stomach disorders (4). Mixed with other plants and given to treat animals suffering from asthma (122). Stem with other formulation is given orally to cattle against endoparasites (26). Decoction of the plant is used to get relief from pain by massaging locally (30). Plant given to domestic animals suffering from fever and dysentery (68).
121	*Cymbidium goeringii* f. *albiflorum* (S.S.Ying) S.S.Ying	Herb	Orchidaceae		Roots	Paste applied to set dislocated bones of leg (31).
122	*Cymbidium elegans* var. *elegans*<br>**Cymbidium longifolium* D. Don	Herb	Orchidaceae		Roots	Boiled root juice fed to livestock suffering from cold (92).
123	*Cymbopogon flexuosus* (Nees ex Steud) W. Watson.	Herb	Poaceae	Chel (Nepali), Yukpa (Tamang)	Leaves	Given as good fodder to naks and cows to enhance lactation (4).
124	*Cynanchum auriculatum* Royle ex Wight	Climber	Apocynaceae	Kauwaloli (Bantar)	Seeds	Used to treat nipple infection (bleeding while drawing out milk) (40).
125	*Cynodon dactylon* (L.) Pers.	Herb	Poaceae	Dubo (Nepali)	Whole plant	Used as veterinary medicine (39). Whole plant is fed to livestock twice a day to enhance the lactation; paste is made by

						crushing the whole plant and applied in a wounded area (26).
126	*Cyperus difformis* L.	Herb	Cyperaceae	Mothe Jhar (Nepali)	Roots	Root nodules are crushed and fed to cure any urinary related problem (26).
127	*Cyperus rotundus* L.	Herb	Cyperaceae		Stem and leaves	Used to treat anthelmintic (115).
128	*Dactylicapnos scandens* (D.Don) Hutch.	Climber	Papaveraceae	Jogi lahara (Nepali)	Aerial parts	Fed to animals as an anthelmentic medicine (71).
129	*Dalbergia sissoo* D.C.	Tree	Fabaceae	Sisau (Nepali)	Leaves	Juice in given in blood dysentery (27). Leaf is crushed to obtain juice, and then mixed with churning curd, and fed to cure diarrhea (26). Leaves are given to cattle to check dysentery and kill intestinal worms (30).
130	*Daphne bholua* Buch.-Ham ex D. Don	Shrub	Thymelaeaceae	Kagatpate (Nepali), Syukudhumbu (Tamang)	Flowers	Juice is put into the nostrils of the yaks to remove the leech during the rainy season (4).
131	*Datura metal* L.	Shrub	Solanaceae	Daturo (Nepali), Dhuthur (Bantar)	Fruits, Leaves	Used to treat diarrhoea (27,40,101) and to treat inflammation (27). Leaf juice used to treat shoulder wound, leaves used to treat joint swelling (27). Fruits are roasted on open fire, and fed to treat diarrhea, dysentery and fever (26). Paste of immature fruit mixed with other ingredients is used to treat diarrhea and dysentery (29). Leaves are toxic to livestock (101). Roasted fruits are given to

						domestic cattle to induce sleep and to check diarrhea (30).
132	*Datura stramonium* L.	Shrub	Solanaceae	Daturo (Nepali)	Seeds	Used to cure indigestion in cattle (74).
133	*Delphinium brunonianum* Royle	Herb	Ranunculaceae	Bisadi ghans (Nepali)	Roots, Leaves	Used in dysentery and ticks in cattle (123).
134	*Delphinium cashmerianum* Royle	Herb	Ranunculaceae		Roots, Leaves	Used to kill intestinal worms and teaks in cattle (3).
135	*Delphinium grandiflorum* L.	Herb	Ranunculaceae	Chharkhang (Tibetan)	Leaves	Used to kill teaks in cattle (3).
136	*Delphinium himalayae* Munz	Herb	Ranunculaceae	Atis, Nirmansi (Nepali), Bhongmar (Tamang)	Roots	Root paste is kept in hoof of animal to expel worms (31,119). It is used to treat diseases in hooves of cattle and also in foot disease in cattle (3). Mixed with other plant and juice is given to treat animals suffering from asthma, roots are also used to kill lice and bed bugs on cattle (122).
137	*Delphinium scabriflorum* D. Don	Herb	Ranunculaceae	Toyoring (Tamang)	Whole plant, Roots, Leaves	Juice from whole plant is used to kill worms in wounds of cattle (1). Juice from root or leaves is used to treat wounds (29). Plant infusion is applied to remove worms from the wounds in the hooves of cattle (31).
138	*Delphinium vestitum* Wall.	Herb	Ranunculaceae	Mauare maulo, Mangra mhul (Magar), Atish, Bikh (Nepali)	Roots	Root juice is given to animal during delivery (73) and food poisoning in cattle (121).
139	*Dendrobium amoenum* Wall. ex Lindl.	Herb	Orchidaceae	Hardjorne (Nepali)	Stem	Crushed and used in bone fracture of cattle (81,116).

140	*Dendrobium longicornu* Lindl.	Herb	Orchidaceae		Roots	Boiled root fed to livestock suffering from coughs (92).
141	*Dendrocalamus strictus* (Roxb.) Nees	Tree	Poaceae	Tama bans (Nepali)	Leaves	Tonic to ailing livestock, but long time feeding act as antigalactgogue (101).
142	*Dendrophthoe falcata* (L. fil.) Blume	Parasite	Loranthaceae		Leaves, Nectar	Leaves paste is applied on fractures for setting bones (64) and livestock worms (50,120). Leaf along with other plant are made into paste and used to treat bone fractures (124).
143	*Desmodium confertum* DC.	Shrub	Fabaceae		Leaves	Given for better lactation (3).
144	*Didymocarpus albicalyx* C. B. Clarke	Herb	Gesneriaceae	Kumkum dhup (Nepali)	Whole plant	Used to energies weak animals (80).
145	*Didymocarpus aromaticus* Wall. ex D.Don	Herb	Gesneriaceae		Whole plant	Juice of plant is fed to domestic animals to treat bovine haematuria (18).
146	*Dimetia scandens* (Roxb.) R.J.Wang<br><br>***Hedyotis scandens*** Roxb.	Shrub	Rubiaceae		Whole plant	Plant is given to enhance secretion of milk (31).
147	*Dioscorea bulbifera* L.	Climber	Dioscoreaceae		Bulb	Juice used as astringent (28).
148	*Dioscorea deltoidea* Wall. ex Griseb.	Climber	Dioscoreaceae	Gittha, Bhyakur (Nepali)	Roots, Tubers	Paste given in diphtheria (69,112). Juice obtained from tuber is given twice a day is to treat constipation (26).

149	*Dioscorea pentaphylla* L.	Climber	Dioscoreaceae	Mithe tarul (Nepali)	Tuber	Crushed mass of tuber is given to cattle when they become sick by eating green leaves of maize. Raw tuber is given to cattle to cure 'vyagute rog' (diphtheria) (94).
150	*Diploknema butyracea* (Roxb.) H.J.Lam<br><br>**Bassia butyracea* Roxb.	Tree	Sapotaceae	Chiuri (Nepali), Singmar (Tamang)	Root, Fruits, Seeds, Bark	Root is crushed to make a paste and fed to cure fever; fruit is crushed and paste obtained is applied to cure mastitis; bark is ground with water and applied by mixing with camphor to cure the wound (26). The crushed ripe fruits are applied externally for skin problems (104). Seed extract is applied on nose of livestock to expel leeches (101). Oil cake is poisonous to chicken (31).
151	*Drimia indica* (Roxb.) Jessop<br><br>**Urginea indica* (Roxb.) Kunth	Herb	Asparagaceae	Ban piyaz (Nepali)	Bulb	Paste is given in fever (27). Tuber paste is given to get relief from stomach swelling and indigestion (30).
152	*Drynaria propinqua* (Wall.ex Mett.) J.Sm. apud Bedd.	Fern	Polypodiaceae	Kamari (Nepali), Nakabhyak (Tamang)	Rhizomes	Paste is applied on bone fracture (4).
153	*Drynaria quercifolia* (L.) J.Sm.	Fern	Polypodiaceae		Whole plant	Plant is hung in the shed to treat or prevent arheya (= 21/2), a disease; killing affected livestock within 21/2 hours (44).
154	*Eclipta prostrata* (L.) L.	Herb	Asteraceae	Bhringraj (Nepali)	Whole plant	Juice is dripped into the eyes of livestock for cleansing purposes and to treat white spots (44). Paste prepared from the whole plant is applied to treat mastitis (28).

155	*Elephantopus scaber* L.	Herb	Asteraceae	Sahasrajari (Nepali)<br><br>Muskur	Roots	Roots are used in pathogens over the wounds of animals (51). Used as sex stimulants in female animals (80). Plant is given to cattle to relieve from dysentery. Roots are used to purify blood (30).
156	*Elettaria cardamomum* (L) Maton	Herb	Zingiberaceae		Seeds	Used to treat blood in urine with other plants (27). Powder is made by crushing dry seeds and fed to animal to cure fever (26).
157	*Eleusine coracana* (L.) Gaertn.	Herb	Poaceae	Kodo (Nepali)	Seeds	Seeds are boiled in water and mixed with cornflour meal to cure chronic cough and any weakness (26).
158	*Elsholtzia flava* Benth.	Herb	Lamiaceae	Ban silam (Nepali)	Roots, Leaves	Roots or the leaf paste is applied to the affected parts to cure scabies in cattle (2).
159	*Entada gigas* (L.) Fawc. & Rendle<br><br>**Entada scandens* L.	Climber	Fabaceae	Pangra (Nepali)	Seeds	Paste used as plaster to join the factured bones in cows (101).
160	*Ephedra gerardiana* Wall. ex Klotzsch & Garcke	Shrub	Ephedraceae	Somlata (Nepali)	Stem	Used to treat cough (23).
161	*Equisetum ramosissimum* Desf.<br><br>***Equisetum debile* Roxb. ex Vaucher	Herb	Equisetaceae	Kurkure ghans (Nepali), Migolja (Tamang)	Whole plant, Stem	Paste is applied over the dislocated bones of the cattle (4). Paste obtained from stem is applied externally on fractured bone (26).
162	*Eragrostis nigra* Nees ex Steud.	Herb	Poaceae	Phurke khar (Nepali)	Whole plant	Plant is given as a nutritious feed (31).
163	*Erythrina arborescens* Roxb.	Tree	Fabaceae	Aejingo, Phaledo (Nepali)	Bark	Extract is anthelmintic and skin abraison (41).

164	*Erythrina stricta* Roxb.	Tree	Fabaceae	Phaledo (Nepali)	Leaves, Bark	Leaf juice is given as vermifuge for cattle (52). Bark is soaked in water and applied over the body to treat scabies (26). Used to treat chronic disease of cattle (61). Bark juice used to treat wounds and foot and mouth diseases (29).
165	*Eulaliopsis binata* (Retz.) C. E. Hubb.	Herb	Poaceae	Babiyo (Nepali), Bankasi (Magar)	Young leaves, whole plant	Used to cure weakness of cattle (91); plant is burnt and the ash with other ingredients is applied to treat cuts and wounds of domestic animals (90). Leaves are feed to cattle to treat indigestion (53). Grass is given raw to cure weakness (26).
166	*Euphorbia hirta* L.	Herb	Euphorbiaceae		Whole plant	Paste applied to treat wounds on shoulder of an ox or a buffalo, caused by yoke; it is also used for the wounds on the back of a horse (29).
167	*Euphorbia royleana* Boiss.	Tree	Euphorbiaceae	Seudi (Nepali), Desya (Tamang), Rijh	Stem, Sap	Plant juice is fed mixed with cattle's food to get rid of intestinal worms (4). Stem is roasted, cooled and given in blood dysentery (27). Milky juice is used to treat Corneal opacity or keratoconjunctivitis (28). Stem is roasted and given to elephants to control diarrhea (30).
168	*Euphorbia wallichii* Hook.f.	Herb	Euphorbiaceae	Dhude jhar (Nepali)	Sap	Plant is poisonous to cattle (29).
169	*Fagopyrum esculentum* Moench	Herb	Polygonaceae	Phapar (Nepali)	Seeds	Seed flour used to treat cataract and retention of placenta (23).

170	*Fagopyrum megacarpum* Hara<br>**Eskemukerjea megacarpum* H. Hara.	Herb	Polygonaceae	Somlata (Nepali), Kre (Tamang)	Leaves	Used in loss of appetite in cattle (121). Leaves crushed with the roots of the *Rumex nepalensis* are prepared into paste and the paste is applied over the area of dislocated bone (4).
171	*Falconeria insignis* Royle<br>**Sapium insigne* (Royle) Trimen	Tree	Euphorbiaceae	Khirro (Nepali), Khalung (Tamang)	Roots, Leaves, Sap	Juice is given in case of stomach disorders of cattle (89) and to kill worms of horns of animals (111).
172	*Ficus auriculata* Lour.	Tree	Moraceae	Nimaro (Nepali)	Fruits	Given to cure the bleeding of cattle with urine locally called "laumate" (81,116).
173	*Ficus benghalensis* L.	Tree	Moraceae	Bar (Nepali); Bargad (Thraru), Banyan tree (English)	Bark	Bark used for livestock worms (88,95,120). Juice used to treat diarrhea (29).
174	*Ficus neriifolia* Sm.	Tree	Moraceae	Dhudhilo (Nepali)	Bark, Leaves	Bark juice used to treat conjunctivitis; leaves are fed as a galactagogue (29).
175	*Ficus religiosa* L.	Tree	Moraceae	Pipal (Nepali), Pipar (Tharu)	Bark	Used in foot and mouth disease (27,40) and rheumatism (54). Used to treat blood in urine with other plants (27). Dry bark is ground to obtain powder and applied to the burn area (26). Bark is used to reduce fever in cattle (30).
176	*Ficus racemosa* L.<br>**Ficus glomerata* Roxb.	Tree	Moraceae	Khasreto (Nepali)	Leaves	Ingested to livestock to ease the expulsion of placenta (101).
177	*Ficus semicordata* Buch.-Ham. ex J. E Sm.	Tree	Moraceae	Khanyu (Nepali)	Leaves	Leaves as fodder are given to remove the placenta (26,115).
178	*Ficus* sp.	Tree	Moraceae	Bedulo (Nepali)	Leaves	Forage acts as galactogogue (101).

179	*Foeniculum vulgare* Mill.	Herb	Apiaceae	Madhise sunp (Nepali)	Leaves	Used to treat indigestion/anorexia (23).
180	*Fragaria nubicola* Lindl.<br>**Potentilla nubicola* (Hook. f.) Mabb.	Herb	Rosaceae	Bhuin ainselu (Nepali)	Whole plant	Used in foot and mouth disease of cattle (123).
181	*Fraxinus floribunda* Wall.	Tree	Oleaceae	Lankuri (Nepali), Kipsil (Tamang), Himalayan ash (English)	Bark	Used to treat stomach disorder, broken arms and legs of cattle (20). Juice extracted from the bark is given to treat the stomachache for the cattle (4). Bark is ground to make powder to cure diarrhea; paste is applied to treat broken legs and arms of animal (26). Juice is given to treat the stomach disorder of sheep, young shoots are toxic (31).
182	*Galium asperifolium* Wall.	Herb	Rubiaceae	Chitu (Nepali)	Whole plant	Juice is given for cataract of cattle, goats, etc. (121).
183	*Gaultheria trichophylla* Royle	Herb	Ericaceae	Nilo bhuikaphal (Nepali), Dabrami (Tamang)	Leaves	Dried leaf powder is applied over the old wounds and to remove the worms from the wound (4)
184	*Girardinia diversifolia* (Link) Friis	Shrub	Urticaceae	Allo (Nepali)	Aerial parts	Nutritious for cows and buffaloes and increases milk production (77).
185	*Gonostegia hirta* (Blume) Miq.	Herb	Euphorbiaceae		Whole plant	Used to treat sprain and facture (105).
186	*Grewia multiflora* Juss.	Tree	Tiliaceae	Phosre (Nepali)	Leaves	Fed as galactogogue (101).

187	*Gynandropsis gynandra* (L.) Briq.<br><br>**Cleome gynandra* L.	Herb	Cleomaceae	Tori jhar (Nepali)	Leaves	Paste applied to treat fresh wound of goat, sheep and other small animals (29).
188	*Helicteres isora* L.	Tree	Malvaceae	Pharsa (Nepali)	Roots, Leaves	Juice from roots and leaves are fed in the morning to cattle suffering from fever and from infections with roundworms (55). Root paste mixed with water is taken to treat worm infestations in livestock; large amounts of tender leaves are fed to livestock to treat worm infestations (44).
189	*Herpetospermum darjeelingense* (C. B. Clarke) H. Schaef. & S. S. Renner	Climber	Cucurbitaceae	Jangali karela (Nepali), Tangsarkato (Tamang)	Seeds	Pounded seeds are mixed with corn flour and are fed to the cattle to relieve the fever (4).
190	*Heterodermia diademata* (Taylor) D.D. Awasthi	Lichen	Physciaceae		Whole plant	Powdered and applied externally on fresh cuts and wounds (125).
191	*Hibiscus sabdariffa* L.	Shrub	Malvaceae		Fruits	Infusion given to treat diarrhea and dysentery (69).
192	*Hippolytia dolichophylla* (Kitam.) K. Bremer & Humphries<br><br>**Tanacetum dolichophyllum* (Kitam.) Kitam. ex Kitam. & Gould	Herb	Asteraceae		Roots	Roots given in indigestion (23).
193	*Hippophae salicifolia* D. Don	Shrub	Elaeagnaceae	Chugo (Nepali)	Fruits	Fruits used as anthelmintic (3,23).

194	*Holarrhena pubescens* (Buch.-Ham.) Wall. ex G. Don	Tree	Apocynaceae	Indrajau (Nepali)	Bark	Plant used as veterinary medicine (39). Decoction obtained from bark is given to the livestock to treat constipation, diarrhea, and dysentery (26).
195	*Hordeum vulgare* L.	Herb	Poaceae		Young leaves, Seed	New leaves are crushed to obtain juice and used to cure eye-related problems; seeds are boiled in water and the oily material is fed orally to treat mastitis (26).
196	*Houttuynia cordata* Thunb.	Herb	Saururaceae	Gane (Nepali)	Whole plant	Juice of plant is put in the wounds of cattle to kill worms and to hasten the healing. 18).
197	*Hydrocotyle sibthorpioides* Lam.<br><br>**Hydrocotyl* sp.	Herb	Apiaceae	Khochade, Sano ghortapre, Zupha Hathi paila, timir jhar (Nepali)	Leaves	Juice is given to livestock for urinary problems (88).
198	*Hymenidium benthamii* (Wall. ex DC.) Pimenov & Kljuykov<br><br>**Pleurospermum benthamii* (DC.) C.B.Clarke	Herb	Apiaceae	Ganyaino (Nepali)	Roots	Used in colic (23).
199	*Hymenidium dentatum* (Wall. ex DC.) Pimenov & Kljuykov<br><br>**Pleurospermum dentatum* (DC.) C. B. Clarke	Herb	Apiaceae		Whole plant	Mixed with other plant and juice is given to treat animals suffering from asthma (122).

200	*Hypericum cordifolium* Choisy	Shrub	Hypericaceae	Areto (Nepali)	Leaves	Tender leaves are poisonous to cattle (86).
201	*Ichnocarpus frutescens* (L.) W. T. Aiton	Shrub	Apocynaceae	Khegar dhudi (Tharu)	Leaves	Fed to animal as galactagogue (47).
202	*Ilex dipyrena* Wall.	Tree	Aquifoliaceae	Seto khasru (Nepali), Bomsi (Tamang)	Leaves	Fresh leaves are fed to the cattle to enhance the secretion of milk and to increase strength and vigor (4).
203	*Impatiens scabrida* DC.	Herb	Balsaminaceae	Phatakya (Nepali)	Whole plant	Plant is given to treat the fever (31).
204	*Imperata cylindrica* (L.) P.Beauv.	Herb	Poaceae	Siru (Nepali)	Aerial parts, Roots	Plant is used for fodder purposively to kill worms and to treat diarrhea in cattle and goats (73). Roots are crushed to make a paste and used to cure endoparasite (26).
205	*Ipomoea himalayana* J. R. I. Wood & Scotland<br><br>**Argyreia hookeri* C.B. Clarke	Climber	Convolvulaceae	Suntiki laharo (Nepali)	Whole plant	Tied externally circling the stomach of an animal to expel placenta (28).
206	*Jatropha gossypiifolia* L.	Shrub	Euphorbiaceae	Rato sajyun (Nepali)	Roots	Paste applied to treat dislocated bones, decoction is given for the same (29).
207	*Juglans regia* L. var. *kamaonia* C. DC.	Tree	Juglandaceae	Okhar (Nepali)	Bark	Paste with another plant is applied to broken and dislocated bones for animals, particularly goats (122).
208	*Juniperus recurva* Buch.-Ham. ex D. Don	Tree	Cupressaceae	Dhupi (Nepali)	Leaves	Used to treat external parasites (23).
209	*Justicia adhatoda* L.	Shrub	Acanthaceae	Asuro (Nepali)	Whole plant, Leaf, Flower	Decoction prepared from the whole plant is given to cure of common cold (26). Leaf

						and flower paste used to treat cough and bronchitis (29).
210	*Justicia pectinata* L.<br><br>******Rungia pectinata* (L.) Nees	Herb	Acanthaceae		Stem	Stem piece tied on body of goat to ease delivery (65).
211	*Kalanchoe spathulata* DC.	Herb	Crassulaceae	Ajamari jhar (Nepali)	Stem, Leaves	Plant is poisonous to cattle (29,96).
212	*Lecanthus peduncularis* (Wall. ex Royle) Wedd.	Herb	Urticaceae	Gakaleti (Nepali), Tilo (Tamang)	Aerial parts	Fresh plant is fed to cattle to stimulate the secretion of milk (4,31).
213	*Lens culinaris* Medik	Herb	Fabaceae	Masuro (Nepali)	Seeds	Cooked with maize flour and given to animal twice a day to increase milk production (26).
214	*Leonurus japonicus* Houtt.	Herb	Lamiaceae	Chhotka bakain (Batar)	Leaves	Used in constipation and tiredness (40).
215	*Lepisorus mehrae* Fraser-Jenk.	Fern	Polypodiaceae		Rhizomes	Powder used to treat diarrhea and wound on mouth (108).
216	*Leucas lavandulifolia* Sm.<br><br>**Leucas indica* (L.) R. Br. ex Vatke	Herb	Lamiaceae	Dhulphe jhar (Nepali), Dulpha (Bantar)	Leaves	Used in urinary problems (stopped urination) (40) and as laxative (54). Juice given in flatulence (27).
217	*Lindera neesiana* (Wall.ex Nees) Kurz.	Tree	Lauraceae	Siltimur (Nepali)	Fruits, Seeds, Leaves	Fruits used to treat animal diseases (85), diarrhea (80). Seeds are given to check diarrhoea and dysentery (4). When cattle eat poisonous plants, dry fruits given (31,73), and also to remove placenta during delivery (73). Fruit juice is given to cure any weakness (26). Fruit juice is used to treat stomach disorder of livestock if

						they eat poisonous plants (106). Fruits medicinal (108). Leaves grinded and given to treat animal disorders (109). Powder or paste is given to treat indigestion (112). Fruits used to remove lice and other ectoparasites (29).
218	*Linum usitatissimum* L.	Herb	Linaceae	Aalus (Nepali)	Seeds	*Linum usitatissimum* and *Sesamum indicum* are fried and crushed to make powder. It is then fed orally to cure mastitis. Similarly, powder obtained from dried seed of *Linum usitatissimum*, *Sesamum indicum* and *Trachyspermum ammi* are mixed and fed with cornflour twice a day to increase milk production (26). Used to treat gastritis and cough and cold (105).
219	*Litsea cubeba* (Lour.) Pers.	Tree	Lauraceae	Siltimur (Nepali)	Seeds	Seeds used in colic (23).
220	*Litsea doshia* (D. Don) Kosterm.	Tree	Lauraceae		Leaves	Fed to treat sicknesses (109).
221	*Luculia gratissima* (Wall.) Sweet	Shrub	Rubiaceae	Ban kangiyo (Nepali), Somrati (Tamang)	Bark	Juice is used to remove worms inside the stomach of cattle (4,31,96).
222	*Luisia tristis* (G. Forst.) Hook. F.<br>**Luisia zeylanica* Lindl.	Herb	Orchidaceae		Leaves	Juice used to treat wounds in cattle (31,70).
223	*Lygodium flexuosum* (L.) Sw.	Fern	Lygodiaceae		Whole plant	Used as veterinary medicine (39).

224	*Lyonia ovalifolia* (Wall.) Drude	Tree	Ericaceae	Angeri (Nepali)	Leaves	Young leaves are smeared, or their juice applied regularly to the affected part to treat scabies in cattle (2). Young leaves are poisonous to cattle (1,48,29, 85,101,118).
225	*Lysimachia arvensis* subsp. *arvensis*<br>******Anagallis arvensis* L.	Herb	Primulaceae	Armale	Whole plant	Plant juice used to remove leeches (29).
226	*Maesa chisia* Buch.-Ham. ex D. Don	Shrub	Primulaceae	Bilauni (Nepali)	Fruits, Leaves	Fruits are given to sterile female buffalo/cow (97). Plant used as veterinary medicine (39). Leaves are given to increase secretion of milk (31).
227	*Maesa macrophylla* Wall. ex Roxb.	Shrub	Myrsinaceae	Bhogte (Danuwar)	Fruits	Juice given to treat haemorrhagic septicemia of animals (46) and diptheria of cattle (31).
228	*Mallotus philippensis* (Lam.) Muell. Arg.	Tree	Euphorbiaceae	Sindhure, Kamala, Rohini (Nepali), Kampillak, Rugnang (Raji); Pyongla (Tamang)	Bark, seeds, fruits	Used to cure gastric trouble of cattle (91).
229	*Malva kashmiriana* Alef.<br>**Lavatera cachemiriana* Cambess.	Herb	Malvaceae	Majaino (Nepali)	Leaves	Used to treat agalactia (23).
230	*Malva verticillata* L.	Herb	Malvaceae	Laphe saag (Nepali)	Whole plant	Plant is boiled and given to cattle for fast expulsion of placenta (3).

231	*Mangifera indica* L.	Tree	Anacardiaceae	Aanp (Nepali)	Bark, Fruits	Bark used to treat urinary problems (40). Paste of dried unripe fruits is given to cattle as an antidote (48). Used to treat eye problems (80). Bark is dried then ground with camphor and applied to the burn area (26).
232	*Marsdenia tenacissima* (Roxb.) Moon	Climber	Apocynaceae	Bilajor (Nepali)	Root	Paste is applied on swelling bone area (26).
233	*Medicago sativa* L.	Herb	Fabaceae	Kosheghans (Nepali)	Leaves	Enhance metabolism and increase milk production in livestock (60).
234	*Melia azedarach* L.	Tree	Meliaceae	Bakaino (Nepali); Aristha, Nimbah (Sanskrit), Chanyal (Tamang), Bakain (Tharu); Bokom bana (Satar); Persian lilac (English)	Fruits, Leaves, Bark	Fruits are useful in *tila pareko* of cattle (128). Decoction from leaves is fed to treat intestinal worms (27). Bark and leaves juice fed to treat intestinal worms (28). Seeds and leaves are crushed to make juice. It is applied by mixing with turmeric and salt to cure ectoparasite; *Melia azedarach*, *Azadirachta indica*, and *Artemisia indica* are taken and cut into pieces. It is cooked with cornflour then feed to treat endoparasite; Seed and leaf are crushed to make a paste and applied to cure wound by mixing with camphor (26).
235	*Mesosphaerum suaveolens* (L.) Kuntze<br>**Hyptis suaveolens* (L.) Poit.	Herb	Lamiaceae	Ban bawari (Nepali)	Leaves	Juice is dropped to treat conjunctivitis (29).

236	*Millettia extensa* (Benth.) Baker	Shrub	Fabaceae	Gaujo (Nepali)	Roots, Leaves	Used to get rid of lice from cattle (96). Leaves are used to treat lice and worms (56). Plant used as veterinary medicine (39). Roots are used in ticks and mites of cattle (51). Plant juice used to treat scabies (28). Root decoction applied on cattle's sore (67). Leaf juice used to treat lice or any other ectoparasites (29,31).
237	*Mimosa pudica* L.	Herb	Fabaceae	Lajjawati jhar (Nepali)	Whole plant	Fed to cure uterus prolapse (26). Used as galactogogue (101).
238	*Mentha arvensis* L.	Herb	Lamiaceae	Pudina (Nepali)	Leaves, Whole plant	Fed with *Allium sativum* to cure diarrhea (26). The whole plant is rubbed into the body of the animal to cure ectoparasite (26).
239	*Mirabilis jalapa* L.	Herb	Nyctaginaceae	Lankeshwori (Nepali)	Roots	Juice used to treat urinary disorders (28).
240	*Moringa oleifera* Lam.	Shrub	Moringaceae	Setakchini (Nepali)	Roots, Bark	Root juice used to treat wounds (69). Bark decoction given as diuretic (27).
241	*Morus alba* L.	Tree	Moraceae	Kimbu (Nepali)	Latex	Used as anthelmintic for cattle (75).
242	*Morus australis* Poir.	Tree	Moraceae	Ban kimbu (Nepali)	Roots	Fresh root is crushed to make a paste and fed to animal for treating endoparasite (26).
243	*Mucuna pruriens* (L.) DC.	Climber	Fabaceae	Kauso (Nepali)	Leaves	Given to animals in case of snake bite (22).
244	*Murraya koenigii* (L.) Sprengel	Shrub	Rutaceae	Karipatta (Nepali)	Leaves	Used to treat lice, ticks and mites of cattle (51).

245	*Musa paradisiaca* L.	Herb	Musaceae	Kera (Nepali)	Root/Stem	Juice given as diuretic (27). Ripen banana is given to treat mastitis (26). Juice of the plant is given to cattle to induce urination (30).
246	*Myristica fragrans* Houtt.	Tree	Myristicaceae	Jaifal (Nepali)	Seeds	Powder is obtained from seeds of this plant, *Trachyspermum ammi*, and *Sesamum indicum* then fed to cure chronic cough, constipation. Solely it is given to increase milk production and urinary problems (26).
247	*Nastus borbonicus* J.F.Gmel. **Bambusa arundinacea* Bonpl.	Tree	Poaceae		Leaves	Fresh leaves fed to cattle for placental retention with quicker parturition (27).
248	*Nicotiana tabacum* L.	Herb	Solanaceae	Surti (Nepali)	Leaves, Bark	Paste is used to remove lice (106) and to cure skin diseases of cattle (98). Juice applied to kill and remove leeches from the nose of cattle (75). Leaves used to treat germs and wounds (40) and external parasites (23). Bark decoction used to treat fever; paste is used to treat wounds (29).
249	*Nyctanthes arbor-tristis* L.	Tree	Oleaceae		Fruits	Used in foot and mouth disease (40).
250	*Ocimum basilicum* L.	Herb	Lamiaceae	Palhas (Bantar), Babari (Nepali)	Leaves	Used to treat germs and wounds (40).
251	*Ocimum tenuiflorum* L. **Ocimum sanctum* L.	Herb	Lamiaceae	Tulsi (Nepali)	Leaves	Used to treat cough (23). Leaf paste is applied externally to healing wounds (26).

252	*Ophiocordyceps sinensis* (Berk.) G.H. Sung, J.M. Sung, Hywel-Jones & Spatafora<br><br>***Cordyceps sinensis*** (Berk.) Sacc.	Fungus	Ophiocordycipitaceae		Whole body parts	Given to cattle as tonic and aphrodisiac (116).
253	*Oreocnide frutescens* (Thunb.) Miq.	Shrub	Urticaceae	Pleta (Gurung)	Roots	Juice is given to cattle to clear the stool (31).
254	*Oreoseris gossypina* (Royle) X. D. Xu & V. A. Funk<br><br>**Gerbera gossypina* (Royle) Beauv.	Herb	Asteraceae		Roots	Used to treat internal parasite (Liver fluke, worms) (23).
255	*Oroxylum indicum* (L.) Kurz	Tree	Bignoniaceae	Tatahalo (Nepali)	Bark, Seeds	Bark of this plant with *Schima wallichii* are dried, then grounded to obtain powder, and fed to cure diarrhea (26). Fresh leaves and seeds are grounded to make a paste, and fed to cure urinary problem (26). Paste used to treat wounds (29).
256	*Osbeckia stellata* Buch.-Ham. ex D. Don	Shrub	Melastomataceae	Angaru, Anger, Asare phul (Nepali), Mendorumrum (Tamang)	Roots, Flowers, Leaves, Fruits	Paste is prepared from the roots and flowers and is applied over the swellings of the dislocated bone (4). A decoction of the leaf and fruit twice a day is given to domestic animals for any kind of weakness (26). Root dectoction is antipoisoinng (31).
257	*Osyris quadripartita* Salzmann ex Decne.	Shrub	Santalaceae		Leaves	Young leaves are poisonous for goat (99).

258	*Osyris wightiana* Wall. ex Wight	Shrub	Santalaceae	Nun dhiki (Nepali)	Bark, Leaves	Dried bark is crushed to make powder and fed to cure joint swelling (26). Leaves are poisonous; bark juice is used to treat indigestion (29).
259	*Oxalis corniculata* L.	Herb	Oxalidaceae	Chariamilo (Nepali), Nakhrupang (Tamang)	Whole plant, Leaves	Plant juice is dropped inside to treat earache of cattle (70). Plant used as veterinary medicine (39). Extract of the fresh leaves is applied over the boils of the domestic cattle (4). Juice used to treat eye complaint (27,31). Juice obtained by pounding fresh leaves is dropped into the eyes for any eye problems (26). Fresh extract leaves is applied to eyes of the domestic cattles to cure eye problem (30). Plant juice is used to treat muscular swelling (31).
260	*Oxyria digyna* (L.) Hill	Herb	Polygonaceae	Ragatmudo (Nepali)	Whole plant	Used in swelling/internal wound (23).
261	*Papilionanthe teres* (Roxb.) Schltr.<br>**Vanda teres* Lindl.	Herb	Orchidaceae		Whole plant	Paste is applied to treat dislocated bones of cattle (45).
262	*Paris polyphylla* Sm.	Herb	Melanthiaceae	Satuwa (Nepali)	Roots, Rhizomes	Used as antidote (99). Root juice is given as an anthelmintic (4). Pieces of root fed with cattle feed to treat diarrhea (107). Roots medicinal (108).
263	*Parthenocissus semicordata* (Wall.) Planch.	Climber	Vitaceae	Charchare lahara (Nepali)	Aerial parts	Given to increase lactation and appetite (29).

264	*Persicaria chinensis* (L.) H. Gross	Herb	Polygonaceae	Kukur thotne (Nepali)	Whole plant	Used as diuretic (80).
265	*Persicaria hydropiper* (L.) Spach	Herb	Polygonaceae	Pirre (Nepali)	Aerial parts	Juice is given to treat worms for cattle (57). Paste is given as anthelmintic (29).
266	*Persicaria nepalensis* (Meisn.) H. Gross	Herb	Polygonaceae	Raune (Nepali)	Whole plant	Plants are poisonous to cattle (119).
267	*Persicaria wallichii* Greuter & Burdet	Herb	Polygonaceae	Ratnaule (Nepali), Yapenguin (Tamang)	Tender parts	Fresh tender parts are given to cattle to enhance the secretion of milk (4).
268	*Phyla nodiflora* (L.) Greene<br>*****Lippia nodiflora* (L.) Michx.	Herb	Verbenaceae		Roots	Paste used to treat wounds, used to treat bone dislocation (29).
269	*Phyllanthus emblica* L.	Tree	Phyllanthaceae	Amala (Nepali)	Leaves and fruits	Paste is made by crushing leaves and fruit and applied to the burn area (26).
270	*Picrorhiza scrophulariiflora* Pennell<br>**Neopicrorhiza scrophulariiflora* (Pennel) D.Y. Hong	Herb	Plantaginaceae	Kutki (Nepali), Hodling (Sanskrit)	Roots	Paste is used to heal the wounds of cattle (2). Mixed with other plant and juice is given to treat animals suffering from asthma (122). Roots used in internal parasites/cough (respiratory problem) (23).
271	*Pieris formosa* (Wall.) D. Don	Tree	Ericaceae	Lekh angeri (Nepali)	Leaves	Plant is poisonous to cattle (1). Leaf paste applied to treat scabies, leaves are poisonous (29).
272	*Pinus roxburghii* Sarg.	Tree	Pinaceae	Sallo (Nepali)	Latex	Latex is mixed with mustard oil and applied to wound skin (26).

273	*Pinus wallichiana* A.B.Jackson	Tree	Pinaceae	Gobre sallo (Nepali)	Resin, stem	Resin/stem used in sprain/internal pain (23).
274	*Piper longum* L.	Climber	Piperaceae	Pipair (Batar), Pipla (Nepali)	Leaves	Used to treat constipation and tiredness (40).
275	*Piper nigrum* L	Climber	Piperaceae	Marich (Nepali)	Fruits, Rhizomes	Fruits used to treat cataract (23). Rhizome paste of this plant with *Zingiber officinale* and *Acorus calamus* is given in fever (27). Fruits powder used to treat chronic cough (27). Powder is prepared and blown to the eye gently to cure eye-related problem (26).
276	*Piptanthus nepalensis* (Hook.) D. Don	Shrub	Fabaceae	Jhyan (Nepali)	Leaves	Juice applied to treat wounds in hoof (29).
277	*Plantago centralis* Pilg.<br>**Plantago erosa* Wall.	Herb	Plantaginaceae	Isabgol (Nepali), Aitanjhar (Tamang)	Whole plant	Extract of whole plant is used for antiseptic dressings (4).
278	*Plantago major* L.	Herb	Plantaginaceae		Roots	Used as anthelmintic (115).
279	*Platostoma hispidum* (L.) A.J.Paton<br>**Acrocephalus indicus* (Burm. f.) Kuntze	Herb	Lamiaceae		Whole plant	Plant used as veterinary medicine (39).
280	*Pleione humilis* (Sm.) D. Don	Herb	Orchidaceae	Bhuisungava (Nepali), Ghabeto (Magar)	Bulb	Used as galactogogue for livestock (88).

281	*Plumbago zeylanica* L.	Herb	Plumbaginaceae	Chit	Roots, Seeds	Juice to treat falling of hairs, paste is given in case of diarrhea (29). Root paste is given orally to reduce effects of snake and scorpion bites. Paste of seeds is given in stomach pain (30).
282	*Pogostemon benghalensis* (Burm. f.) Kuntze	Herb	Lamiaceae	Rudilo (Nepali), Utajar	Leaves, Flowers, shoots	Juice is mixed with old about a year cow-dung and given to cattle to control dysentery (31,58). Plant used in veterinary medicine (39). Leaf and flower are dried in sun. Powder is prepared by crushing them and fed to cure diarrhea (26). Leaf and flower are crushed to make a paste, and applied to cure a wound (26). Boiled and given in case of cough and bronchitis (29). Paste of fresh shoots is applied to wounds and bandaged (30).
283	*Polyalthia longifolia* (Sonn.) Thwaites	Tree	Annonaceae		Leaves	Paste applied in eye problem of cattle (19).
284	*Polygonatum verticillatum* (L.) All.	Herb	Asparagaceae		Roots	Paste given to dogs as a health tonic in debility (31).
285	*Polygonum* sp.	Herb	Polygonaceae		Young shoots	Used to cure dysentery (115).
286	*Portulaca oleracea* L.	Herb	Portulacaceae	Nona, Nunia	Leaves	Decoction of leaf is given to cattle to stop urinal bleeding, improves urination (30).
287	*Potentilla indica* (Andr.) Wolf<br>**Duchesnea indica* (Andrews) Focke	Herb	Rosaceae	Bhui Kaphal (Nepali)	Roots	Fed to treat colic in cattle (81).

288	*Premna barbata* Wall. ex Schauer	Shrub	Lamiaceae	Gineri (Nepali)	Leaves	Juice is sprayed on fowl, cattle to remove fleas ("Sulsule") (69).
289	*Prunus armeniaca* L.<br><br>**Prunus nepalensis* Hort. ex C. Koch	Tree	Rosaceae	Jsangali aaru (Nepali)	Bark, Leaves	Juice is applied to kill ectoparasites, leaves are poisonous to cattle (29).
290	*Prunus buergeriana* Miq.<br><br>*****Prunus venosa* Koehne	Tree	Rosaceae	Chari aare (Nepali)	Leaves	Leaves are poisonous, paste applied to treat swelling (29).
291	*Prunus cornuta* (Wall. ex Royle) Steud.	Tree	Rosaceae	Ban aaru (Nepali)	Bark	Used to treat livestock wounds (128).
292	*Prunus davidiana* (Carrière) Franch.	Tree	Rosaceae		Bark	Juice is applied externally to kill ticks in cattle (3).
293	*Prunus persica* (L.) Stokes	Tree	Rosaceae	Aaru (Nepali), Tokpo (Lepcha)	Leaves, New tips	Juice of leaf is put to take out any worms inside the wounds of animals (71). New tips are put in pathogens over the wounds of cattle (51). A paste with another plant is applied to broken and dislocated bones for animals, particularly goats (122). New leaf buds are taken and crushed to make juice. Then it is mixed with camphor and applied when endoparasites are seen. It is also applied to treat wound (26). Used to treat foot and mouth disease (63). Paste or juice from young shoot is used to treat cut and wounds (112).
294	*Psidium guajava* L.	Tree	Myrtaceae	Latam (Bantar), Amba (Nepali)	Leaves, Bark	Tender leaf used to treat urinary problems (stopped urination) (40). Leaves paste applied locally to treat wounds of animals; raw or grinded bark mixed with animal

						feed is given to the animal to control diarrhea (116).
295	*Punica granatum* L.	Tree	Lythraceae	Anar (Nepali)	Bark	Paste is given to domestic cattle to get relief from asthma (30).
296	*Pyrus pashia* Buch.-Ham. ex D. Don	Tree	Rosaceae	Mael (Nepali), Ghorphal (Magar)	Bark, fruit	Grinded fruits flour is given to cattle for more milking (88,119,126); the filtered juice of unripe fruit is used in cattle eye to cure white spot (48). Fruits used in constipation/ cataract (23). Plant juice used to treat eye troubles (111,117).
297	*Rauvolfia serpentina* (L.) Benth. ex Kurz	Shrub	Apocynaceae	Sarpagandha (Nepali)	Roots	Root decoction was given to cattle to cure diarrhea (31,58). Root paste used to treat fever and stomachache (41).
298	*Rhaphidophora decursiva* (Roxb.) Schott	Climber	Araceae	Kanchirno (Nepali)	Whole plant	Plant is given raw or mixed with flour and given to cattle to cure urinary problem (112).
227	*Rheum australe* D. Don	Herb	Polygonaceae	Padamchal (Nepali)	Roots, Stem	Root paste is applied on the injured or broken limbs for cattle (3,116). Root and stem used to treat sprain and facture (23).
300	*Rhododendron anthopogon* D. Don	Shrub	Ericaceae		Aerial parts	Plant odor is considered anti-poisoning (108).
301	*Rhododendron campanulatum* D. Don	Tree	Ericaceae	Chimal (Nepali)	Leaves	Poisonous to cattle (29,119).
302	*Ricinus communis* L.	Shrub	Euphorbiaceae	Ander (Nepali); Aril, Rend, Raine (Tharu); Renu (Magar), Iradam (Satar); Dandarobi	Seeds, Leaves, Flowers	Leaf powder is used to cure wounds for animal (22). Paste is made from the seeds and is applied over the swollen parts due to cold (4). The flowers are pounded with

				(Tamang), Castor oil plant (English)		water and the paste obtained is applied to treat skin problems of cattle (104).
303	*Rochelia zeylanica* (Vahl) Roem. & Schult.<br><br>**Cynoglossum zeylanicum* Thumb. ex. Lehm.	Herb	Boraginaceae	Bhere kuro (Nepali)	Whole plant	Powder obtained from dried plant is blown to eye problem; paste of plant mixed with water and applied to cure skin scabies and wound (26).
304	*Rubia manjith* Roxb. ex Fleming	Climber	Rubiaceae	Majitho (Nepali), Tiru (Tamang), Indian madder (English)	Whole plant, Stem	Useful in inflammation of cattle (128). It is also given to cattle to expel placenta (3). Stem used in liver disease (23).
305	*Rubia wallichiana* Decne.	Climber	Rubiaceae		Stem	Useful in inflammation of cattle (128).
306	*Rubus ellipticus* Sm.	Shrub	Rosaceae	Aiselu	Leaves	Leaves are crushed to make a paste and applied in the wound area (26).
307	*Rumex nepalensis* Spreng.	Herb	Polygonaceae	Halhale (Nepali), Hali (Gurung), Sheep sorrel (English)	Roots, Young shoots, whole plant	Used as antidote for food poisoning (22). Root paste is applied over the dislocated bone and is also applied with frame of bamboo stick at the bone fracture segment until relief (4). Plant is boiled to treat diarrhea of a goat (29). Young shoots are given to cattle for energy (116). Root decoction is used to treat diarrhea of the cattle (31).
308	*Saccharum officinarum* L.	Herb	Poaceae	Ukhu (Nepali)	Stem, Leaves	Stem is crushed to obtain juice, boiled, and fed by mixing with cornflour to increase milk production; leaf is given as feed to remove placenta (26).

309	*Saccharum spontaneum* L.	Herb	Poaceae	Kaans (Nepali)	Roots	Paste is directly applied to remove helminths of cattle (75).
310	*Salix denticulata* Andersson	Tree	Salicaceae		Leaves	Poisonous to sheep (29).
311	*Salvia plebeia* R. Br.	Herb	Lamiaceae		Leaves	Rubbed externally on feet to remove lice of cattle (22).
312	*Sapindus mukorossi* Gaertn.	Tree	Sapindaceae	Ritha (Nepali)	Fruit	Fruit with its covering are cooked with cornflour and water to cure urinary problem (26).
313	*Sarcococca coriacea* (Hook.) Sweet<br>**Sarcococca pruniformis* Lindl.	Shrub	Buxaceae	Phitphiya (Nepali), Gherpati (Tamang)	Seeds	Powdered seeds are mixed with corn flour to feed orally to treat the stomachache (4).
314	*Saurauia napaulensis* DC.	Tree	Actinidiaceae	Gogan (Nepali)	Leaves	Used to cure cattle dysentery and gastric (113).
315	*Schefflera venulosa* (Wight & Arn.) Harms	Shrub	Araliaceae		Leaves	Given as feed to treat the conjunctivitis of cattle (31,89).
316	*Schima wallichii* (DC.) Korth.	Tree	Theaceae	Chilaune (Nepali); Gwe chhasima, Khyu si (Gurung); Ghyansing (Magar), Chyangsi (Chepang); Sakriphal (Satar);	Bark, Leaves	Given orally to cattle suffering from Taeniasis (91); Bark juice with other ingredients is given to relieve stomach disorders of cattle (89), given to animals infested with liver flukes (86) and also as an anthelmintic (4,31,71). Pounded bark is given to animals to treat liver fluke (87). Bark paste given to animal to treat namle disease (100). Powder used to treat stomach disorders and liver fluke (28).

				Kyasing (Tamang)		Bark of this plant and *Oroxylum indicum* are dried, then ground to obtain powder, and given orally to cure diarrhea. Given to cure endoparasite (26). Powder is medicinal (108). Bark juice used to treat cough (29). Decoction of bark and leaves is given to get rid of intestinal worms of cattle (30).
317	*Scindapsus officinalis* (Roxb.) Schott	Herb	Araceae	Kanchirno (Nepali)	Fruits	Given to treat stomach worms (31,62). Boiled and dropped to treat eye disease, given to treat diarrhea and also put in wound to remove maggot (29).
318	*Scoparia dulcis* L.	Herb	Plantaginaceae	Chini jahr (Nepali)	Stem	Stem is crushed, and the paste is applied to the swelling area (26).
319	*Scurrula parasitica* L.	Parasite	Loranthaceae	Jokhare (Nepali)	Tender shoots	Given in loss of appetite and vomiting to livestock (101).
320	*Scutellaria scandens* D. Don	Parasite	Lamiaceae		Roots	Juice is given to domestic animals to prevent miscarriage (86).
321	*Semecarpus anacardium* L. fil.	Tree	Anacardiaceae		Fruits	Used to treat cut, paste with *Allium sativum* is used to treat tail cut (27). Fruit is ground with mustard oil and applied to wound (26).
322	*Senegalia catechu* (L.f.) P.J.H.Hurter & Mabb.<br>**Acacia catechu* (L.f.) Willd.	Tree	Fabaceae	Khair (Nepali)	Bark	Bark is given to elephant to check intestinal worms, diarrhea and dysentery; bark decoction is given to treat diarrhea and dysentery to other domestic animals (30).

323	*Senegalia pennata* (L.) Maslin<br><br>**Acacia pennata* (L.) Willd	Tree	Fabaceae	Arari (Tharu)	Bark	Juice is fed to domestic animals to relieve sprain and muscular swellings (18).
324	*Senna tora* (L.) Roxb.<br><br>**Cassia tora* L.	Shrub	Fabaceae	Tapre ,Chakon, Tapre,Sanotapre (Nepali); Ayadham, Chakwad (Tharu), Chakramardha (Sanskrit); Sickle pod (English)	Seeds, Leaves	Used in falling of hair of cattle (58) and fever (41,52,96). Seed powder/paste is taken as potential anthelmintic for cattle (102). Leaf powder is rubbed onto parts of cattle affected by falling of hair (58). Seed powder is given to cattle as an anthelmintic, for immature cattle, sheep and goats, the dose is lowered proportionately (82). Plant used as veterinary medicine (39). Squeezed leaves rubbed on body of animal to check the falling of hairs (29). Roasted seeds are given to animals for a mouth to fatten the animals (30).
325	*Sesamum indicum* subsp. *indicum*	Herb	Pedaliaceae		Seeds	Seeds oil used to treat external wound (23). Powder is obtained from seeds of this plant, *Trachyspermum ammi* and *Myristica fragrans* and then fed to cure chronic cough and constipation. *Sesamum indicum*, *Trachyspermum ammi* and *Linum usitatissimum* are fried and crushed to make powder and fed to cure mastitis and increase milk production (26).
326	*Shangwua denticulata* (DC.) Raab-Straube & Yu J. Wang	Herb	Asteraceae	Bhukur (Nepali), Jhya (Tamang)	Leaves	Leaf extract is given to control the fever; the plant is tied around the tail to stimulate the milk secretion and is practiced to Naks in high altitude (4).

327	*Shorea robusta* Gaertn.	Tree	Dipterocarpaceae	Saal (Nepali), Sakhua	Resin, Stem, Bark	During hoof infection in cattle resin is mixed with oil and applied on the hoof daily (102). Fresh decoction of stem bark is given to check diarrhea (30).
328	*Sida acuta* Burm. f.	Herb	Malvaceae	Balu, Kuro (Nepali)	Roots	Used as buti in the sickness of cattle (69).
329	*Sida cordata* (Burm. fil.) Borss. Waalk.	Herb	Malvaceae	Kuro (Nepali)	Whole plant	Plant paste is out in mouth of cattle if the milching is suddenly stopped (29).
330	*Sida rhombifolia* L.	Herb	Malvaceae	Balu jhar; Thulo balu, Bishkapre, Sano chilya, Sano khareto (Nepali)	Leaves	Paste used in breast encouragement (69).
331	*Sigesbeckia orientalis* L.	Herb	Asteraceae		Whole plant	Plant used as veterinary medicine (39).
332	*Solanum lycopersicum* L.	Herb	Solanaceae	Tamatar (Nepali)	Fruit	Fruit is crushed, and then the juice is applied to the burn area; fruit is directly fed to livestock to cure endoparasite; fruit juice is applied to cure mastitis (26).
333	*Solanum melongena* L.	Herb	Solanaceae		Fruit, Leaves	Roasted fruit paste with mustard oil is used to treat shoulder pain (27). The fruit is roasted and fed to animal to cure diarrhea, new leaves are crushed and fed to remove the placenta (26).
334	*Solanum tuberosum* L.	Herb	Solanaceae		Tubers	Paste is applied to treat burnt wound (27). Tuber is cut and rubbed externally on burns twice a day (26).

335	*Solanum virginianum* L.	Herb	Solanaceae	Rengani	Whole plant, Roots, Fruits	Whole plant including roots is smoked and given to elephant to check diarrhea and intestinal worms. Fruit is eaten raw for relief from cough (30).
336	*Solena amplexicaulis* (Lam.) Gandhi ex Saldanha & Nicolson<br>**Solena heterophylla* Lour.	Climber	Cucurbitaceae	Ban kakri, Golkakri, Bankankro (Nepali)	Whole plant, Roots	Whole plant is used to increase milk production in cattle (22,31); proper development of foetus (31); supplied as feed to she-buffalo or cow to improve lactation (89). Plant used as veterinary medicine (39). Paste used to treat mastitis (28). The paste prepared from the root is applied to cure mastitis; The whole plant is considered a nutritious feed, and also used to increase milk production (26). Root juice is given to kill intestinal worms in cattle (116).
337	*Spermacoce alata* Aubl.<br>**Spermacoce latifolia* Aubl.	Herb	Rubiaceae	Paundhi (Nepali)	Whole plant	Juice applied to heal fractured bone of cattle (31,58).
338	*Spermadictyon suaveolens* Roxb.	Shrub	Rubiaceae	Ban chaap (Nepali)	Bark	Paste is given to treat cough in animals (86).
339	*Sphaeranthus senegalensis* DC.	Herb	Asteraceae	Bonsupari (Nepali), Ganhuwa (Danuwar)	Flowers	Flower heads fed to animals to treat ear troubles (46).
340	*Stellaria monosperma* Buch.-Ham. ex D. Don	Herb	Caryophyllaceae		Roots	Powder is given in case of diarrhea (31).

341	*Stephania glandulifera* Miers	Climber	Menispermaceae	Biral gano, Batulpate, Batule (Nepali); Gudargano (Chepang)	Roots, Whole plant	Used as veterinary medicine (69). Peeled root mixed with corn flour is given to cattle as tonic (70). Juice from whole plant is used to treat stomach disorder (28). Roots given to treat diarrhea (29,31,108).
342	*Stephania gracilenta* Miers	Climber	Menispermaceae	Biralkano (Nepali)	Roots	Used to treat swelling (23).
343	*Stephania japonica* (Thunb.) Miers	Climber	Menispermaceae	Muklak (Nepali)	Roots	Root tubers given to ox as feed supplement (74).
344	*Streblus asper* Lour.	Tree	Moraceae	Khaksi (Nepali), Shihora	Latex, Leaves, twigs	Applied on the eye of the cattle during eye problem (19). Leaves paste used to treat burnt wound (27). Twigs are burnt to ash and used for healing wounds (30).
345	*Strobilanthes tomentosa* (Wall. ex Nees) J. R. I. Wood<br><br>**Aechmanthera gossypina* (Nees) Nees	Shrub	Acanthaceae	Kangaraito phul (Nepali)	Roots	Paste is applied to guard against internal injuries (31).
346	*Swertia chirayita* (Roxb. ex Fleming) Karsten	Herb	Gentianaceae	Chirayito (Nepali)	Roots, Stems	Root/stem used as anthelmintic (23). Useful in throat pain (128).
347	*Swertia ciliata* (D. Don ex G.Don) B.L. Burtt.	Herb	Gentianaceae	Chirayito (Nepali), Hoba (Tamang)	Whole plant	Juice is given to yaks and sheep to relieve from fever (4).
348	*Symplocos paniculata* (Thunb.) Miq.	Tree	Symplocaceae	Lodh (Nepali), Syungen (Tamang)	Leaves	Given to cattle to enhance the secretion of milk (4).

349	*Syzygium cumini* (L.) Skeels	Tree	Myrtaceae	Jamun (Nepali)	Bark	Juice given to treat diarrhea (29).
350	*Syzygium nervosum* DC.<br>**Cleistocalyx operculatus* (Roxb.) Merr. & Perry	Tree	Myrtaceae	Kyamuna (Nepali)	Bark	Plant used as veterinary medicine (39). Bark juice is given to cattle to treat muscular swellings (31).
351	*Tagetes erecta* L.<br>**Tagetes patula* L.	Herb	Asteraceae		Roots	Fresh roots are ground and fed to treat urinary problem (26).
352	*Tamarindus indica* L.	Tree	Fabaceae	Imli, Titri (Nepali)	Bark, Leaves	Infusion of bark along with ingredients is given to cure flatulence of cattle (66). Decoction of mature leaves is applied locally to reduce pain from wound or beating (30).
353	*Taraxacum officinale* Wigg.	Herb	Asteraceae	Tukiphool (Nepali), Bhutil mendo (Tamang)	Roots, leaves, whole plant	Paste of the plant is applied over the dislocated bones and also bound in fractured bones and fixed with bamboo sticks instead of plaster until relief. Root and leaf used for the cough (4).
354	*Taxus wallichiana* Zucc.<br>**Taxus baccata* subsp *wallichhiana* (Zucc.) Pilg.	Tree	Taxaceae	Lauth salla (Nepali)	Leaves	Leaves are poisonous to cattle (29).
355	*Tephrosia purpurea* (L.) Pers.	Herb	Fabaceae	Kande sakinu (Nepali)	Whole plant	Paste applied for wounds and boils (29).
356	*Terminalia alata* B. Heyne ex Roth	Tree	Combretaceae	Saj (Nepali)	Resin	Applied to treat swelling caused by injury (62).

357	*Terminalia bellirica* (Gaertn.) Roxb.	Tree	Combretaceae	Barro (Nepali), Bahera	Fruits	Fresh fruit is given orally to treat diarrhea (26). Fruit is used to control diarrhea for the domestic cattles. Dry fruit powder is given to cattle to improve indigestion (30).
358	*Terminalia chebula* Retz.	Tree	Combretaceae	Harro (Nepali)	Fruit	Fruit is used to control diarrhea for the domestic cattles. Dry fruit powder is given to cattle to improve indigestion (30).
359	*Tetradium fraxinifolium* (Hook. fil.) T. G. Hartley<br>***Evodia fraxiniflora*** (D. Don) Hook. f.	Tree	Rutaceae		Fruits	Paste and decoction used to treat fever, cough and cold, fatigue, stomach pain, dyspepsia (105).
360	*Tetrataenium nepalense* (D. Don) Manden.<br>**Heracleum nepalense* D. Don	Herb	Apiaceae		Whole plant, Fruit	Whole plant is nutritious fodder to increase the milk in cattle, given for better lactation (3). Paste used to treat stomach problem and diarrhea (105).
361	*Tetrataenium wallichii* (DC.) Manden. DC.	Herb	Apiaceae		Whole plant	Given to cattle for better lactation (3).
362	*Thalictrum chelidonii* DC.	Herb	Ranunculaceae	Dampate (Nepali), Urmen (Tamang)	Roots	Juice is given to cows to control the fever (4).
363	*Thalictrum foliolosum* DC.	Herb	Ranunculaceae		Roots, Leaves	Paste of roots and leaves is given to expel intestinal worms in cattle (2,82).
364	*Thysanolaena latifolia* (Roxb. ex Hornem.) Honda<br>***Thysanolaena maxima*** (Roxb.) Kuntz	Shrub	Poaceae	Amriso (Nepali)	Leaves	Indigestion and cough problems in cattle (59). Leaves as a feed are given to remove placenta (26).

365	*Tinospora cordifolia* (Willd.) Miers	Climber	Menispermaceae	Gurjegana (Magar), Gurjo (Nepali); Gurju (Tamang); Gurj, Giloy (Tharu); Bhrun (Raji)	Stem	Given orally to cattle to cure sterility (91). Piece of stem is fed to cattle for their good health and lactation (45). Stem juice given in appetite loss (27). Stem is made into small pieces, then boiled with water and fed to cure chronic cough and constipation, the powder is obtained from dried stem and given orally with water to treat diarrhea (26). Pieces are given in case of cough of cattle (31).
366	*Tinospora sinensis* (Lour.) Miers	Climber	Menispermaceae	Gurujlati (Bantar), Gurjo (Nepali)	Whole plant	Plant used to treat tiredness (40). Tonic to cattle (69). Plant is galactogouge and given to cattle to increase lactation and to treat diarrhea and dysentery (68).
367	*Toxicodendron wallichii* (Hook. fil.) Kuntze<br>**Rhus wallichii* Hook. f.	Tree	Anacardiaceae	Bhakimlo (Nepali)	Fruits	Used to treat constipation (23).
368	*Trachyspermum ammi* (L.) Sprague	Herb	Apiaceae		Seeds	Powder given in flatulence and constipation (27). Given to animals by mixing with cornflour to cure constipation and increase milk production (26).
369	*Trichosanthes cucumerina* L.	Climber	Cucurbitaceae	Chichindo (Nepali)	Seeds	Dried seeds are crushed to make powder and fed by mixing with turmeric to cure endoparasite (26). Fruit is fed raw to treat mastitis and removing the placenta (26).
370	*Trichosanthes cucumerina* subsp. *anguina* (L.) Greb.	Climber	Cucurbitaceae	Chichindo (Nepali)	Fruits	Powder used to treat mastitis (28).

	Trichosanthes anguina L.					
371	*Trichosanthes tricuspidata* Lour.	Climber	Cucurbitaceae	Indreni, Inddrayani (Magar)	Fruits, roots	Used in boils, abdominal pain, gastritis of cattle (99,103), Fruit is considered as digestive and applied in paralysis, gastric and tila of cattle (128).
372	*Tridax procumbens* L.	Herb	Asteraceae	Thikuri (Danuwar)	Whole plant	Mixed with animal feed to treat hemorrhagic septicemia of animals (46).
373	*Trifolium repens* L.	Herb	Fabaceae		Whole plant	Given to cattle for better lactation (3).
374	*Trigonella foenum-graecum* L.	Herb	Fabaceae	Methi	Seeds	Soaked seeds are mixed with cornflour meal, and given orally to cure chronic cough and fever; *Trigonella foenum-graecum* and *Zingiber officinale* are mixed and fed to cure joint swelling (26).
375	*Trillium govanianum* Wall. ex D.Don	Herb	Liliaceae	Satuwa (Nepali)	Roots	Used in external wound/poisoning (plant poisons) (23).
376	*Uraria lagopus* DC.	Herb	Fabaceae	Sano bhatte (Nepali), Tewalapte (Tamang)	Roots	Root crushed with turmeric is applied over dislocated bones and bound in fractured bone, bandaged and wrapped with bamboo sticks for one month; it is usually practiced to the cows (4).
377	*Urtica ardens* Link	Herb	Urticaceae	Ghariya sisnu (Nepali), Kultuk (Lepcha)	Root	Paste is applied in sprain (112).

378	*Urtica dioica* L.	Herb	Urticaceae	Sisnu (Nepali), Polo (Tamang)	Tender parts, Fruits	Given to increase lactation (4,76,101). Leaf is boiled, and water is fed to an animal to cure mastitis and urinary problem (26). Fruit paste used to treat sprain in cattle (63).
379	*Vachellia nilotica* subsp. *indica* (Benth.) Kyal. & Boatwr.<br><br>**Acacia nilotica* subsp. *indica* (L.) Willd. ex Delile	Tree	Fabaceae	Babul (Nepali), Babur (Batar)	Bark	Bark used in urinary problem (40).
380	*Verbascum thapsus* L.	Herb	Scrophulariacea e	Bandar puchhre (Nepali), Pangjalu (Tamang)	Roots	Roots of the plant are mixed with the roots of *Urtica dioca* and are prepared in the form of paste and applied over dislocated bones. Paste of the root is also applied on the muscular swelling in cases of phantom effect (4).
381	*Vicia sativa* L.	Herb	Fabaceae	Narkat (Nepali), Nakta (Batar)	Whole plant	Whole plant given for lactation (40).
382	*Vicia sativa* subsp. *nigra* (L.) Ehrh.<br><br>**Vicia angustifolia* L.	Herb	Fabaceae		Whole plant	Plant is poisonous to cattle (67).
383	*Vigna mungo* (L.) Hepper	Herb	Fabaceae	Maas (Nepali)	Whole plant	Preparation applied on broken and dislocated bones of animals supported by wooden frame, tied with either a rope or a cloth (122).
384	*Vigna radiata* (L.) R.Wilczek	Herb	Fabaceae		Seeds	Seed flour used in fracture of limbs/foot and mouth disease (23).

	**Phaseolus radiatus* Linn.					
385	*Viscum album* L.	Parasite	Viscaceae	Ainjeru (Nepali)	Whole plant, Bark, Stem	Plant paste is applied to treat dislocated bones and wounds in cattle (20,70).Used to treat various diseases (80). The stem is crushed and paste is applied to the swelling area. It can also be fed by mixing with ghee and honey (26). Bark paste is given in case of muscular swellings and is also used to treat boils and wounds (31).
386	*Vitex negundo* L.	Shrub	Lamiaceae	Senwar	Leaves, Seeds	Decoction used to treat joint swelling (27). Boiled extract of the leaf is applied on scabies and other skin infections in animals (26). Decoction of leaves is given to cattle to get relief from rheumatism. Paste of tender shoots is applied locally to get relief from scorpion bite (30). Crushed seeds are given to cure cough and cold of animals (31).
387	*Woodfordia fruticosa* (L.) Kurz	Shrub	Lythraceae	Dhayero (Nepali)	Flowers	Plant used as veterinary medicine (39).
388	*Xanthium strumarium* L.	Herb	Asteraceae	Bhende kuro (Nepali)	Leaves	Paste applied to treat wound of a goat, sheep and other small animals (29).
389	*Zanthoxylum armatum* DC.	Shrub	Rutaceae	Timur (Nepali)	Fruits	Fruits used to expel leeches from nostrils (23).
390	*Zanthoxylum oxyphyllum* Edgew.	Shrub	Rutaceae		Fruits	Used to kill lice and teaks (80).
391	*Zea mays* L.	Herb	Poaceae	Makai (Nepali)	Smut	Head smut infected corn is crushed by mixing with lemon juice and fed to treat diarrhea; Maize flour is made from corn

						seed and boiled with salt and water to increase milk production (26). Corn flour is mixed with *Asparagus racemosus* root juice and the mixture is given to buffaloes to stimulate milk production (104).
392	*Zingiber officinale* Roscoe	Herb	Zingiberaceae	Kachur (Nepali)	Rhizomes	Foot and mouth diseases and other communicable diseases (80). Used as appetizer (23). Rhizome paste of this plant with *Piper nigrum* and *Acorus calamus* is given in fever (27). Raw rhizome is crushed and given to cure diarrhea, mastitis. It is crushed with *Trigonella foenum-graecum* to treat joint swelling; Raw rhizome is ground with camphor to treat wounds (26). Rhizome paste is given in cough and fever (29).
393	*Ziziphus mauritiana* Lam.	Tree	Rhamnaceae	Bayer (Nepali)	Bark	Decoction used to treat wounds on shoulder of an ox or a buffalo, caused by yoke (29).
